# High-Accuracy Identification of Incident HIV-1 Infections Using a Sequence Clustering Based Diversity Measure

**DOI:** 10.1371/journal.pone.0100081

**Published:** 2014-06-12

**Authors:** Xia-Yu Xia, Meng Ge, Jenny H. Hsi, Xiang He, Yu-Hua Ruan, Zhi-Xin Wang, Yi-Ming Shao, Xian-Ming Pan

**Affiliations:** 1 The Key Laboratory of Bioinformatics, Ministry of Education, School of Life Sciences, Tsinghua University, Beijing, China; 2 The State Key Laboratory for Infectious Disease Prevention and Control, National Center for AIDS/STD Control and Prevention, Chinese Center for Disease Control and Prevention, Beijing, China; Centro Nacional de Microbiología - Instituto de Salud Carlos III, Spain

## Abstract

Accurate estimates of HIV-1 incidence are essential for monitoring epidemic trends and evaluating intervention efforts. However, the long asymptomatic stage of HIV-1 infection makes it difficult to effectively distinguish incident infections from chronic ones. Current incidence assays based on serology or viral sequence diversity are both still lacking in accuracy. In the present work, a sequence clustering based diversity (SCBD) assay was devised by utilizing the fact that viral sequences derived from each transmitted/founder (T/F) strain tend to cluster together at early stage, and that only the intra-cluster diversity is correlated with the time since HIV-1 infection. The dot-matrix pairwise alignment was used to eliminate the disproportional impact of insertion/deletions (indels) and recombination events, and so was the proportion of clusterable sequences (*P_c_*) as an index to identify late chronic infections with declined viral genetic diversity. Tested on a dataset containing 398 incident and 163 chronic infection cases collected from the Los Alamos HIV database (last modified 2/8/2012), our SCBD method achieved 99.5% sensitivity and 98.8% specificity, with an overall accuracy of 99.3%. Further analysis and evaluation also suggested its performance was not affected by host factors such as the viral subtypes and transmission routes. The SCBD method demonstrated the potential of sequencing based techniques to become useful for identifying incident infections. Its use may be most advantageous for settings with low to moderate incidence relative to available resources. The online service is available at http://www.bioinfo.tsinghua.edu.cn:8080/SCBD/index.jsp.

## Introduction

Accurate estimates of HIV-1 incidence are essential for monitoring transmission dynamics, designing and evaluating intervention programs, and optimizing resource allocation [1]–[3]. However, the long asymptomatic stage of HIV-1 infection makes it difficult to effectively distinguish incident (recent) infections from chronic (pre-existing) ones. Direct measurement of incident HIV-1 infections through longitudinal cohort studies is expensive, time-consuming, and subject to observational biases due to selective recruitment and follow-up attrition [4]. The widely used Serological Testing Algorithm for Recent HIV Seroconversion (STARHS), a category of laboratory techniques using an antibody testing approach, are cheaper, quicker and simpler to implement at the population level [5]–[8], but face limitations in terms of standardization, reproducibility, and HIV-1 subtype dependence [9]–[11]. Thus, there is a strong need to develop alternative measurements of incident HIV-1 infections [12].

It is known that the intra-patient HIV-1 viral diversity gradually builds up through time: diversity increases in an approximately linear mode for the first several years after infection, then at decreasing rates until a plateau is reached, and often declines in the late stage of infection [13]–[15]. Recently, two studies have found success in designing accurate incidence assays using such viral genetic diversity measures. Park *et al*. used the 10% quantile (Q_10_) of the pairwise Hamming distance (HD) and achieved greater than 96.0% accuracy when tested on a dataset containing 225 samples [16]. As well, Yang *et al*. developed a pattern-based method and achieved 94.6% accuracy using a dataset of 424 samples [17].

The main difficulties of using viral diversity to determine HIV-1 incidence are in correctly identifying incident infections with multiple transmitted/founder (T/F) strains and chronic infections with declined viral genetic diversity [16]–[18], as well as in reducing the disproportional impact of insertion/deletions (indels) and recombination on calculated diversity value. At the early phase of HIV-1 infection, distinct T/F strains would evolve into highly homologous sequence clusters with relatively low intra-cluster and high inter-cluster diversity [19], [20]. Since the infection duration is only correlated with intra-cluster diversity, overall comparisons of the mean diversity are likely to misclassify early infections with multiple T/F strains. Indeed, while the HD Q_10_ and pattern-based methods were able to conquer this obstacle well for incident infections with two T/F strains [16], [17], their performances were both much reduced for cases involving three or more T/F viruses. Moreover, the *env* gene region of HIV-1 experiences high rates of indels and recombination, and diversity measures based on simple sequence alignment and pattern matching may be disproportionally impacted [21]. Efforts at improving sequence based incidence assays must therefore seek to address these shortfalls.

In the present work, we developed a novel intra-patient diversity based method to determine HIV-1 incident infections. Through utilizing the fact that viral sequences derived from distinct T/F strains tend to form separate homologous clusters, and that only the intra-cluster diversity is correlated with time since HIV-1 infection at the early phase, we created a sequence clustering based diversity (SCBD) metric that clearly and accurately identified incident infections from either single or multiple T/F strains. We employed a modified dot-matrix pairwise comparison method to eliminate the disproportional impact of indels and recombination, and used the proportion of clustered sequences (*P_c_*) as an index to identify late chronic infections with declined viral genetic diversity.

## Materials and Methods

### Dataset Construction

We searched the Los Alamos HIV database (http://www.hiv.lanl.gov/; last modified on February 8, 2012) for all SGA sequencing based samples with ≥5 sequences (median, 26 sequences; range, 5 to 166) containing the *env* gene gp120 C2-V5 region (HXB2: 7050–7590). All samples were obtained from blood serum, plasma, or peripheral blood mononuclear cells (PBMC) of HIV-1 infected individuals. The resulting dataset of 561 samples, named D561, contained all 225 samples used by Park *et al.* (referred to as D225) and 336 others. In total, D561 comprised 12,778 *env* SGA sequences obtained from 462 individuals.

All cases were identified as incident or chronic infections using combined information from the original sources, including Fiebig stage [22], clinical records of time since diagnosis (incident infection defined as under one year), and/or symptoms of acute infection. Thus, 398 of the 561 samples were determined by their sources as incident infections (including 103 cases of multiple infections), with 228 subtype B infections from 196 individuals, 136 subtype C from 99 individuals, and 34 others; the remaining 163 samples are chronic infections, comprising 96 subtype B infections from 96 individuals, 57 subtype C from 49 individuals, and 10 others. The routes of exposure included 257 samples (163 incident and 94 chronic) from 231 heterosexual transmissions; 127 samples (81 incident and 46 chronic) from 109 men who have sex with men (MSM); 41 samples (31 incident and 10 chronic) from 34 intravenous drug users (IDU); and 136 unknown ones. Most of the samples were from treatment naive individuals, except for 9 that were collected from individuals on or after antiretroviral therapy (ART). Detailed information of D561 is listed in the Table S1.

### Sequence Alignment and Diversity-based Clustering

**Figure 1:**
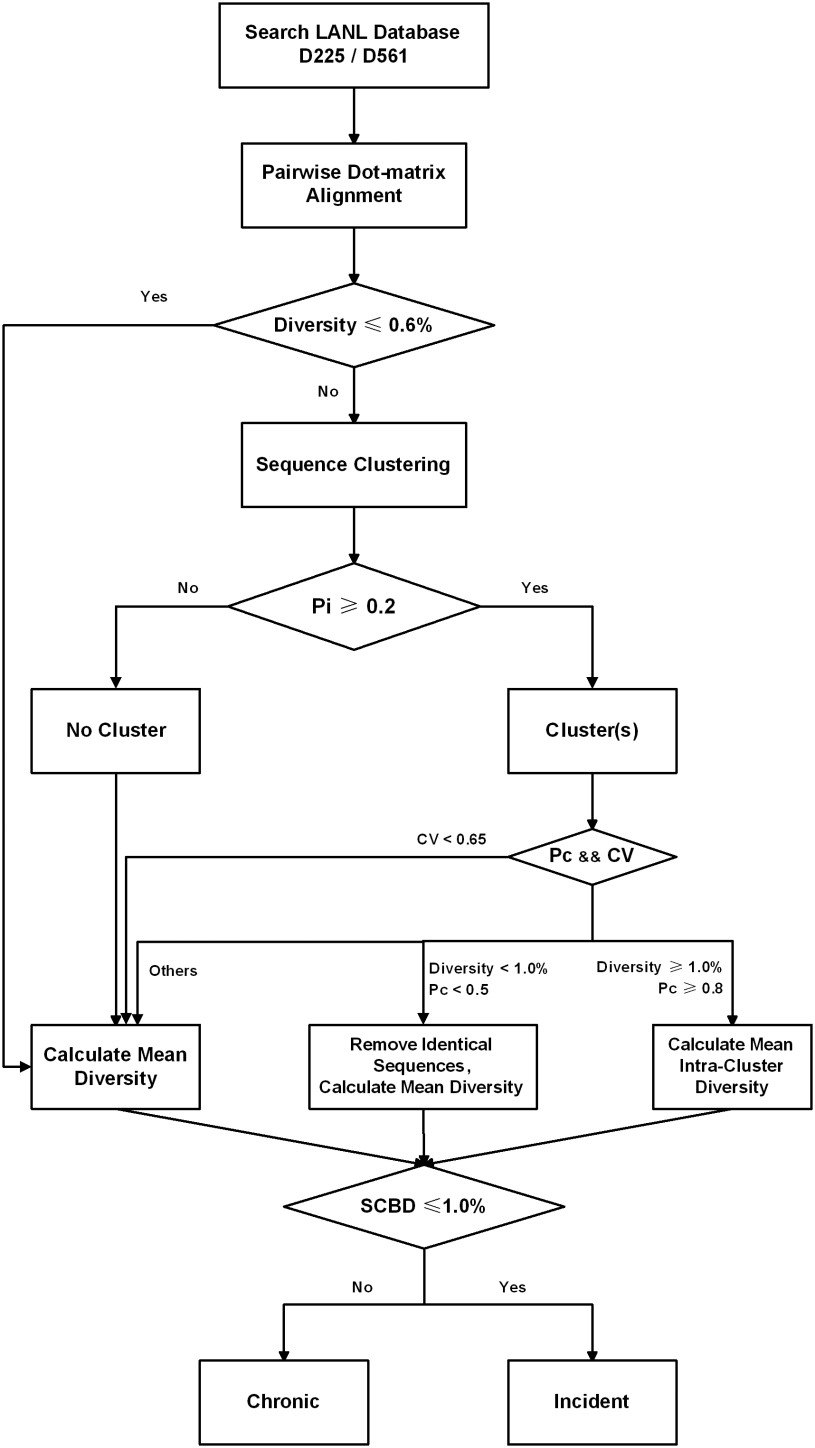
Flowchart for identifying incident from chronic HIV-1 infections using the sequence clustering-based diversity (SCBD) assay.

During multiple HIV-1 infection, viruses arisen from the same T/F strain tend to display lower diversity compared with those delineated from other T/F strains, particularly in early infection [19], [20]. We therefore performed clustering of viral sequences for each sample, and defined the SCBD value as the metric for distinguishing incident and chronic infections, as represented in the flowchart (Figure 1).

First, pairwise sequence alignments were performed for all samples in the dataset. Simple measures of pairwise evolutionary distance, such as Hamming distance, relied on alignment methods that did not take into account the nature of the evolutionary events experienced, and can sometimes inflate the calculated diversity [23]. We therefore used iterative dot-matrix alignment, followed by a modified dynamic programming method to find the optimal pairwise alignment. Briefly, two sequences were written along the top row and leftmost column of a two-dimensional matrix, placing a dot where only if continuous 10 nucleotide matches occurred. The longest diagonal separating the sequence pair into unaligned segments was collected, while a segment pair was defined as an interval if the length of either segment was within the window size of 50 nucleotides. The 10 nucleotide match criterion captured most of the non-random nucleotide matches during modeling at mutation rates up to 10% (the upper limit for the HIV-1 *env* gene), while the 50-nucleotide window size was set to capture at least one indel or recombination event in the unaligned segment pair. The unaligned segments were then iteratively analyzed using above steps, until no diagonal could be found. An interval whose two segments were of equal length was analyzed base by base to detect point mutations; an interval segment pair of unequal length was considered to be the result of an indel or recombination event.

Previous studies have determined that the mean pairwise sequence diversity of the HIV-1 *env* gene region increases at a roughly constant rate of ∼1.0% per year during early infection, with a standard deviation of ∼0.2% [13]. Thus, we defined an incident infection as having occurred within one year to date and a chronic infection as having occurred more than one year ago, and calculated the pairwise sequence diversity and the overall mean value for each sample. If this mean diversity was less than 0.6% (the lower bound of the 95% confidence interval for an incident infection’s diversity value), it was directly recorded as the sample’s SCBD. Otherwise, sequences were gradually grouped into clusters such that pairwise diversity for every sequence pair within the cluster was less than 1.0% (equivalent to the diversity at one year of infection). Only clusters that contain more than 20% of the sample’s total sequences and three or more sequences each were retained for further consideration, as most multiple infections of HIV-1 originate from approximately 2 to 5 T/F strains [19], [20].

Additionally, it has been suggested that pairwise diversities of early infections originating from single T/F strain follow a Poisson distribution [20], with equal mean diversity and standard deviation values and hence a *CV* value (standard deviation/mean diversity) of 1.0. In contrast, the *CV* value of early infections caused by multiple, diverse T/F strains is expected to be higher than 1.0 and descends quickly as time goes on [17]. To correctly recognize chronic infections, we set a cutoff *CV* value of 0.65; this approximates the limit of *CV* in a chronic sample after 2 years of infection with two equally sized clusters. For any case with a lower *CV* value, the overall mean diversity (*D_overall_*) was recorded as its SCBD value.

Furthermore, as some late-stage infections exhibit a decline in the overall viral diversity [13], [14], care must be taken to distinguish such cases from incident infections [18]. Since the viral sequences in these late-stage infections have experienced longer evolutionary times and have undergone reproductive selection, their sampled sequences are mostly unable to form clusters, while those from incident multiple infections are mostly able to. We thus supplemented the calculation of SCBD values using the following metric. Using *N_i_* to denote the number of sequences in the *i*th cluster, and *N_t_* as the total number of sequences in the sample, we calculated the clusterable proportion, or *P_c_*_,_ of each sample as:
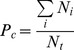
(1)


Clusters were considered valid only when *P_c_*≥0.8 for a sample with mean diversity greater than 1.0%, and the averaged value of the intra-cluster mean diversities were recorded as the SCBD values. Clusters were considered not valid when *P_c_*≤0.5 for a sample with mean diversity lower than 1.0%, and identical sequences were removed from each sample, followed by calculation of the SCBD value using the remaining sequences. Otherwise, the SCBD value was calculated using all sequences.

### Binary Classification of Incident and Chronic Infections

We defined the SCBD cutoff value as 1.0%, equivalent to the result of one year of HIV-1 viral evolution [13], and classified infections as either incident or chronic based on the calculated SCBDs. The sensitivity, specificity, and the overall accuracy for the dataset were calculated respectively. The Mathew Correlation Coefficient (MCC) value [24], which is related to the chi-square statistic for a binary classification, was also calculated as a quality measure of the classification scheme. To compare the performance of our method with those of the existing sequence-based approaches, we also evaluated the performance metrics of the HD Q_10_ method with a cutoff value of 0 as suggested by Park *et al* [16], as well as the pattern-based method with 0.32 as suggested by Yang *et al* [17].

## Results

### Homologous Clusters of Cases in Datasets D225 and D561

We performed pairwise sequence comparison, *D_overall_* calculation, and highly homologous sequence clustering for all cases in D561, including the full set of 225 cases from Park *et al* [16]. As shown in Table 1, 152 incident cases of D225 and 327 of D561 (∼80%) had *D_overall_* lower than 0.6%, which could be easily distinguished from the chronic ones. Among them, most were infections with a single T/F virus (136 of D225 and 282 of D561), while the others were originated from two or more closely homologous T/F strains (16 of D225 and 45 of D561), according to the original resources. The other 30 incident cases of D225 and 71 ones of D561 with higher than 0.6% diversity were mainly infections with multiple T/F strains (30 of D225 and 58 of D561), of which our method further identified ∼75% of such cases (21 of D225 and 43 of D561) as incident infections containing distant homologous clusters. The remaining few incident cases were determined to consist of one major homologous cluster. On the other hand, the great majority of the chronic cases could not be clustered (43 of D225 and 155 of D561), indicating that sequences had greatly diverged over time.

**Table 1:** Homologous clusters identification for cases in datasets D225 and D561.

Dataset	Clusters*	*D_overall_* ≤0.6%			*D_overall_* >0.6%		
		Incident**		Chronic	Incident**		Chronic
		Single	Multiple		Single	Multiple	
D225	S	136	16	0	0	9	0
	M	0	0	0	0	21	0
	D	0	0	0	0	0	0
	-	0	0	0	0	0	43
	Total	136	16	0	0	30	43
D561	S	282	45	1	10	15	0
	M	0	0	0	3	43	3
	D	0	0	0	0	0	4
	-	0	0	0	0	0	155
	Total	282	45	1	13	58	162

*Homologous clusters identified by the SCBD assay.

‘S’: single; ‘M’: multiple; ‘D’: declined; ‘-’: null.

**Incident infections were identified either as ‘single’ or ‘multiple’ infected according to resource information.

### Sequence Clustering Based Diversity (SCBD) Distribution

**Figure 2:**
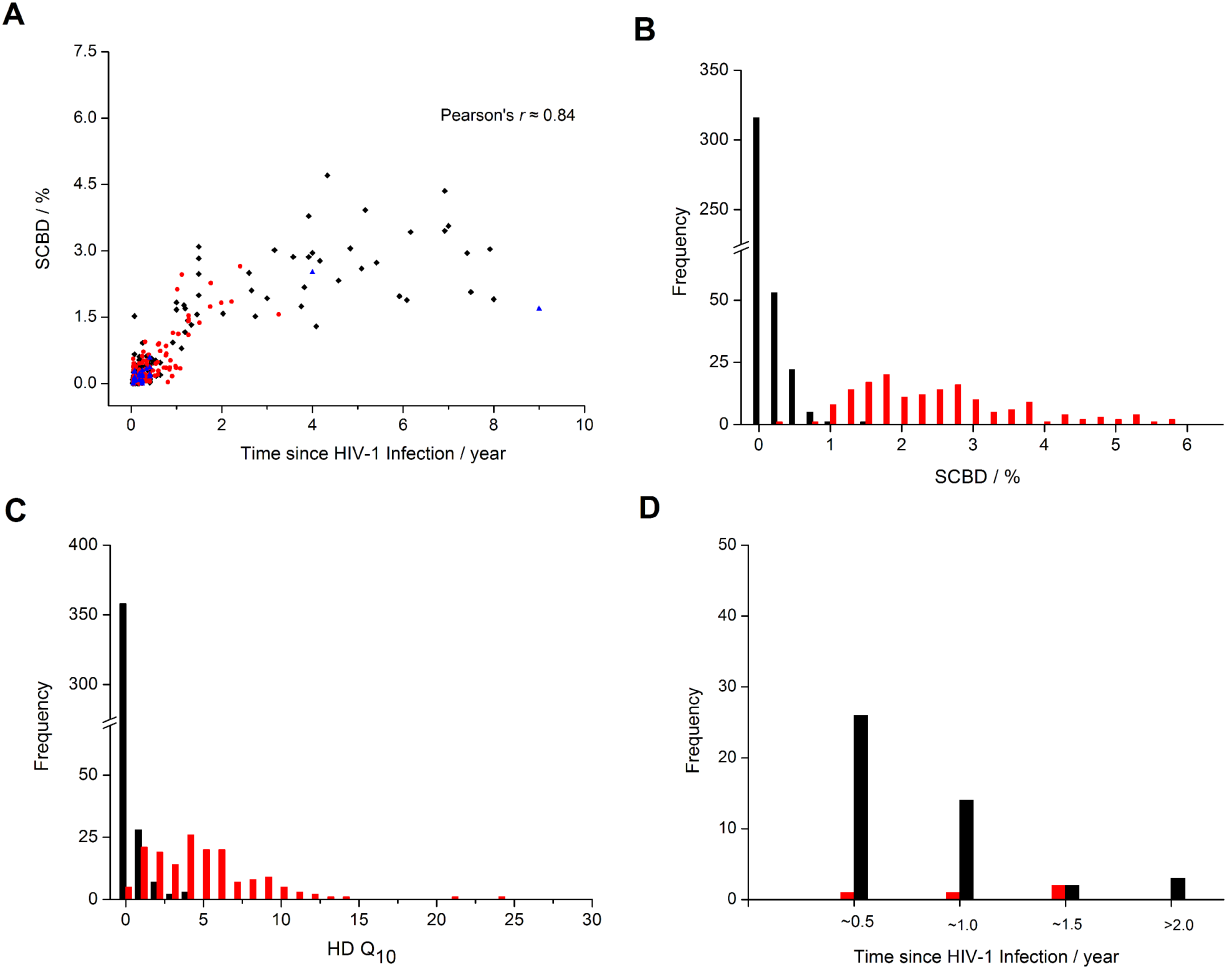
The SCBD dynamics and distribution of D561. (A) The SCBD dynamics in D561. Black diamonds denote subtype B samples, red dots denote subtype C samples, and blue triangles denote samples with other or unknown subtypes. (B) The distribution of the SCBD values in D561. The black histogram denotes the 398 incident infections (including 295 single- and 103 multiple-strain infections), and the red represents the other 163 chronic samples. (C) The distribution of the HD Q_10_ values in D561. (D) The red histogram represents the 4 misclassified samples by our SCBD method, including 2 false positive incident infections and 2 false negatives; the black histogram denotes the 45 misclassified samples by the HD Q_10_ method, including 5 false positives and 40 false negatives.

We further calculated the SCBD value for all cases in D561, including the full set of D225. Figure 2A plots the intra-patient HIV-1 SCBD dynamics, and Figure 2B shows the distribution of SCBDs of incident and chronic cases separately. There was a clear distinction between the SCBDs of the 398 incident samples and those of the 163 chronic samples, which allowed us to define a binary classification test using a simple cutoff SCBD value. In contrast, the HD Q_10_ method yielded a high degree of overlap between the diversity distributions of the incident samples and the chronic ones (Figure 2C). This suggested a strong advantage of SCBD over HD Q_10_ as a metric for distinguishing incident and chronic HIV-1 infections.

### Performance on the Datasets D225 and D561

To assess the validity of our SCBD-based classification test, we first examined its performance on the 225-sample dataset employed by Park *et al* [16]. Meanwhile, the results were compared with those of the HD Q_10_, and our previous pattern-based methods. As shown in Table 2, our method correctly classified all 225 samples, while the HD Q_10_ method misclassified 7 of the 182 incident infections as chronic ones, and the pattern-based method misclassified 3 incident and 4 chronic infections. Cross-checking with source information revealed that 6 of the 7 samples misclassified by the HD Q_10_ method, and all 3 incident samples misclassified by the pattern-based method were from multiple-infected individuals, confirming the particular weakness of both the HD Q_10_ (*p*<0.01, χ^2^ test) and pattern-based (*p* = 0.02, χ^2^ test) methods in correctly identifying the source of sequence diversity in multiple incident infections (Table 2). The SCBD method, in contrast, surmounted this challenge. The difference in classification accuracies between the three methods was statistically significant (*p* = 0.02, χ^2^ test).

**Table 2:** Performance of the SCBD, HD Q_10_, and Pattern-based assays.

Dataset	Assay	Accuracies					
		Sensitivity			Specificity	Accuracy	MCC
		Single	Multiple	Overall			
D225	SCBD	136/136 (100.0%)	46/46 (100.0%)	182/182 (100.0%)	43/43 (100.0%)	225/225 (100.0%)	1.0
	HD Q_10_	135/136 (99.3%)	40/46 (87.0%)	175/182 (96.2%)	43/43 (100.0%)	218/225 (96.9%)	0.91
	Pattern-based	136/136 (100.0%)	43/46 (93.5%)	179/182 (98.4%)	39/43 (90.7%)	218/225 (96.9%)	0.90
D561	SCBD	294/295 (99.7%)	102/103 (99.0%)	396/398 (99.5%)	161/163 (98.8%)	557/561 (99.3%)	0.98
	HD Q_10_	271/295 (91.9%)	87/103 (84.5%)	358/398 (89.9%)	158/163 (96.9%)	516/561 (92.0%)	0.83
	Pattern-based	295/295 (100.0%)	90/103 (87.4%)	385/398 (96.7%)	138/163 (84.7%)	523/561 (93.2%)	0.83

We further tested the performance of our method and those of HD Q_10_ and pattern-based diversity on an expanded SGA-based dataset, D561. As shown in Table 2, our SCBD assay correctly classified all but 2 of the 398 incident infections and 2 of the 163 chronic infections, maintaining a high performance with 99.5% sensitivity, 98.8% specificity, 99.3% overall accuracy and an MCC value of 0.98. In contrast, while the HD Q_10_ method also yielded a high specificity value of 96.9%, it misclassified 40 of the 398 incident infections and yielded a sensitivity value of only 89.9%, bringing the overall accuracy and the MCC value down to 92.0% and 0.83. The pattern-based diversity yielded a high sensitivity value of 96.7%, but misclassified 25 of the 163 chronic infections and yielded a low specificity value of 84.7%, bringing the overall accuracy and the MCC value down to 93.2% and 0.83, which outperformed the HD Q_10_ method but was still much lower than our SCBD method. The difference in performance on this dataset was statistically significant (*p*<0.01, χ^2^ test). Moreover, we continued to observe an advantage in the SCBD method in correctly classifying multiple-strain incident infections: sensitivity values for multiple and single incident infections were 99.0% and 99.7% (*p* = 0.98, χ^2^ test), respectively, compared to 84.5% and 91.9% from HD Q_10_ (*p* = 0.02, χ^2^ test), and 87.4% and 100.0% from the pattern-based method (*p<*0.01, χ^2^ test).

Finally, we assessed the influences of viral subtype and transmission routes on the SCBD assay’s performance. As shown in Table 3, all performance metrics were higher than 98.0% for all subtype categories, including subtypes B, C, and others. While values appeared somewhat lower for subtype C, the differences were not statistically significant (*p* = 0.95, χ^2^ test). Similarly, no statistically significant differences were found between the performance metrics of samples with different transmission routes (*p* = 0.17, χ^2^ test). These results indicated that our SCBD method was robust to virus- and host-specific factors such as viral subtypes and transmission routes.

**Table 3:** Influence of viral subtype and transmission route over the SCBD assay’s performance on the dataset D561.

Factor	Category	Accuracies			
		Sensitivity	Specificity	Accuracy	MCC
Subtype	B	227/228 (99.6%)	95/96 (99.0%)	322/324 (99.4%)	0.99
	C	135/136 (99.3%)	56/57 (98.2%)	191/193 (99.0%)	0.98
	Others	34/34 (100.0%)	10/10 (100.0%)	44/44 (100.0%)	1.0
Exposure	SH	162/163 (99.4%)	94/94 (100.0%)	256/257 (99.6%)	0.99
	MSM	81/81 (100.0%)	46/46 (100.0%)	127/127 (100.0%)	1.0
	IDU	31/31 (100.0%)	10/10 (100.0%)	41/41 (100.0%)	1.0
	Unknown	122/123 (99.2%)	11/13 (84.6%)	133/136 (97.8%)	0.87

### Distribution of Misclassified Cases

The infection time distribution of the cases misclassified by our SCBD and the HD Q_10_ method was shown in Figure 2D. The majority of misclassified cases in HD Q_10_ were incident infections, and the distribution peak was located at 3–6 months. In contrast, most of the misclassified cases (3/4) under the SCBD method had time since infection between 6–18 months, with a nearly equal number of cases on either side of the one-year binary classification threshold.

### Web-Server

To make the method accessible to all laboratory biologists, we have developed SCBD web-server. The server takes a maximum of 500 sampled intra-host SGA viral sequences at a time, and returns information of total number of sequences, clustering status, the SCBD value, and the incident/chronic identification result. Meanwhile, standalone package and the D561 dataset used in the research are also provided for download. The SCBD is freely available at http://www.bioinfo.tsinghua.edu.cn:8080/SCBD/index.jsp.

## Discussion

Lack of accurate ways to identify incident (recent) infection and distinguish them from pre-existing (chronic) ones is one of the major obstacles for measuring HIV-1 incidence, which is essential for monitoring HIV-1 transmission dynamics, designing and evaluating intervention programs, and optimizing resource allocation [1]–[3]. In the present study, we have developed a novel, highly accurate assay for distinguishing HIV-1 incident infections from chronic ones, using intra-cluster viral sequence diversity as the main metric. The SCBD assay clearly identified incident infections, including those originating from multiple T/F strains, at 99.5% sensitivity and 98.8% specificity on the dataset D561; the high performance was robust to factors such as viral subtype and transmission route. Our method therefore brought substantial improvements over existing techniques and indicated significant promise for practical application.

**Figure 3:**
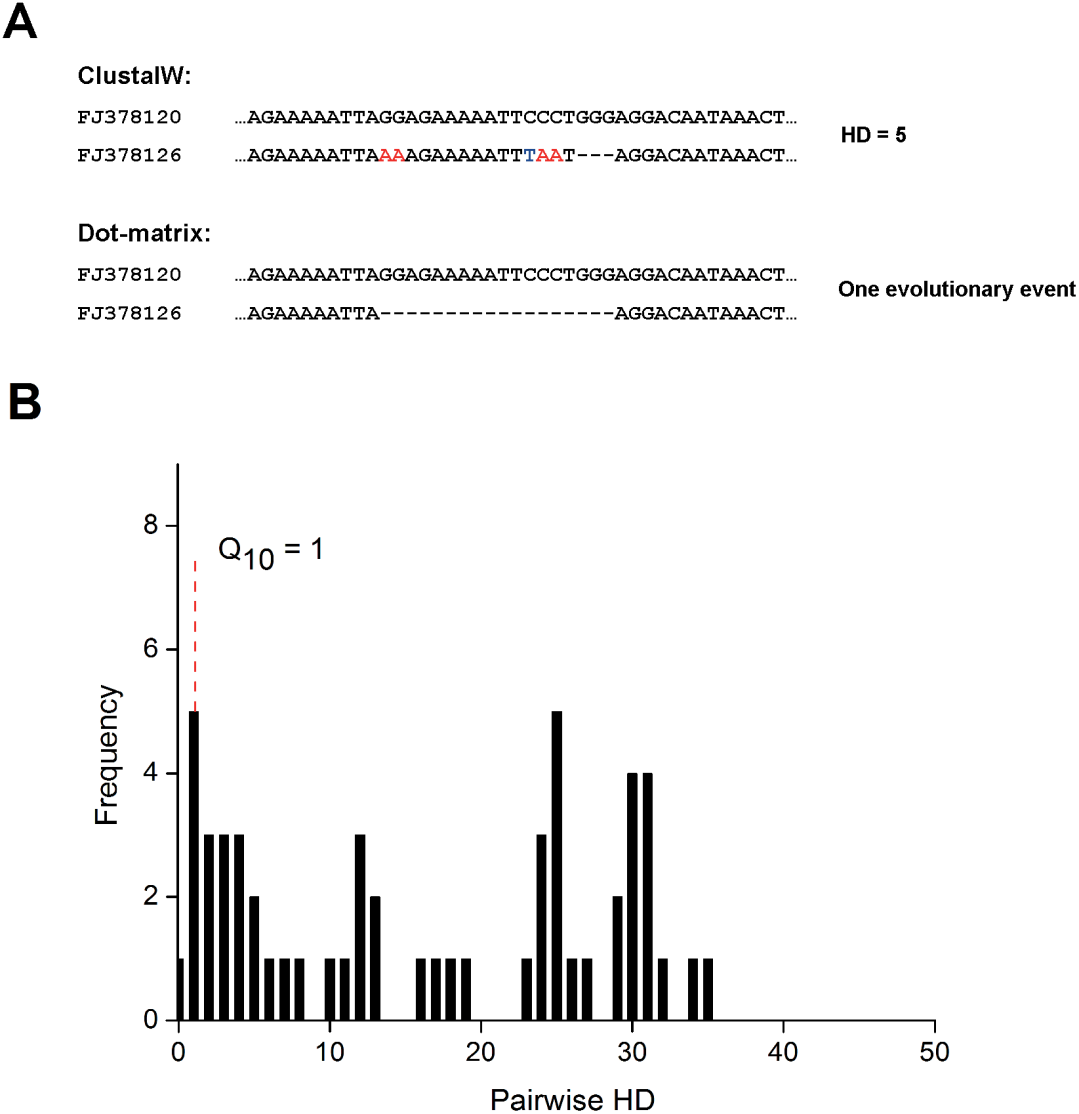
Partial alignment of two sequences from sample ID 1811 (A), along with the sample’s pairwise HD distribution (B). (A) Partial alignment of two sequences from sample ID 1811. Red and blue letters highlight differences in nucleotides. (B) The pairwise HD distribution of sequences from sample ID 1811, which was misclassified as a chronic case by the HD Q_10_ assay (HD Q_10_ = 1). The sample comprised of 11 sequences and was obtained at Fiebig stage VI.

Moreover, previous HIV-1 incidence assays based on viral gene diversity were subject to three main challenges, all of which the SCBD method addressed effectively. First, incident infections involving multiple T/F strains exhibit high inter-cluster diversity that inflates overall diversity measures. While the HD Q_10_ and pattern-based methods appeared to have conquered this obstacle well for infections originating from up to two T/F strains, their performance on three or more T/F strains were far inferior; in contrast, the SCBD method maintained high sensitivity values for both multiple and single incident infections. Secondly, measurements of sequence diversity must also be correct for the inflation in diversity values due to indels and recombination events. For instance, comparing two partial sequences from the sample obtained from patient 1811, their HD value of 5 couldn’t reflect the fact that only one evolutionary event had occurred (Figure 3A); thus, the HD Q_10_ method misclassified this case of multiple incident infection as a chronic case (Figure 3B). The SCBD method, however, accounted for the impact of indels and recombination through the use of dot-matrix alignment, and successfully clustered two clusters with 6 and 3 closely homologous sequences (out of a total of 11), yielding an SCBD value of about 0.31% and correctly identifying the case as incident. Finally, through the clusterable proportion (*P_c_*) and coefficient of variance (*CV*) metrics for identifying chronic infections with decreased diversity, the SCBD method correctly identified two long infected samples from patients SK200 (*P_c_* = 0.3) and SPFE4120 (*P_c_* = 0.28) as chronic, while the HD Q_10_ and pattern-based methods both misclassified these chronic cases as incident.

Among the cases misclassified by the SCBD method, the majority (3 of 4) had estimated time of infection between 6–18 months, with roughly an equal number of cases on either side of the one-year binary classification cutoff (Figure 2D). This is likely due to variations in the evolutionary rates of individual T/F viruses: an incident infection seeded by a fast-evolving strain may give rise to diversity values greater than the 1.0% cutoff within the first year of infection, and vice versa. Previous studies have found that the standard deviation of the HIV-1 *env* gene region’s evolutionary rate is approximately 0.2% during initial infection [13]. Assuming normality, 95% to 99.7% of observed diversity values can be expected to fall within the 0.4–0.6% range on each side of the mean diversity, corresponding to a ∼5 to 7 month window on either side of the one-year mark. This, in fact, agrees well with our results. In contrast, the cases misclassified by HD Q_10_ were mainly incident infections, peaking at the 3–6 month time frame (Figure 2D). The SCBD method can therefore be more reliably employed than methods such as HD Q_10_ in the estimation of incidence on a population level, as the normally distributed false negative and false positive cases may largely compensate each other during rate calculations.

One foreseeable issue of our SCBD assay is the labor intensiveness and high cost of SGA sequencing techniques compared with serology methods, which may limit its large scale application. However, our method offers substantial improvements in assay specificity and sensitivity, which may effectively offset its deficits when taken into account the concerns of serological methods in reliability and reproducibility. In addition, technical challenges inherent to SGA have often curtailed the rates of successful amplifications and hence may bring to question the generalizability of diversity-based methods on a population level. However, failed SGA amplifications are typically due to poor preservation of sample nucleic acids, and not tendentious bias towards particular types of samples. At the same time, deep sequencing methods which produce more than 10,000 reads from a single sample lends a highly promising alternative for addressing the problems of cost, bias, and efficiency, and future research should therefore seek to apply the SCBD method on deep-sequence data. As well, the current availability of large sets of Sanger-sequenced data in the public domain on the HIV-1 *pol* gene, with ambiguous base calls preserved for genotypic drug resistance analysis, also holds promise as a basis for diversity-based incidence assays [15], [25].

However, the high performance of our assay demonstrates the potential of sequencing based techniques as a strong alternative for identifying incident HIV infections. Its substantial improvement in assay specificity and sensitivity may effectively counter its material and labor costs as compared with serology methods, particularly in research settings and for areas under intensive incidence surveillance. Consequently, the SCBD method may be most advantageous to settings with low to moderate incidence relative to available resources, where extensive coverage of the targeted or sample population can be achieved through only a small number of sequencing assays [26], [27]. In contrast, SCBD may be more valuable to regions of higher HIV incidence as a validation assay on a subset of all STARHS-screened samples. Strategic employment of the SCBD method in combination with serology based methods will help to efficiently clarify HIV epidemic trends in differently affected areas.

## Supporting Information

Table S1 **Detailed information of the dataset D561.**
(DOC)
