# Supplementary material for: High-Accuracy Identification of Incident HIV-1 Infections Using a Sequence Clustering Based Diversity Measure
Source: PLoS One. 2014 Jun 12;9(6):e100081. doi: 10.1371/journal.pone.0100081 (PMC4055723; doi:10.1371/journal.pone.0100081)
Supplement: Table S1 — Detailed information of the dataset D561. (DOC) [file pone.0100081.s001.doc]

**Table S1. Detailed information of the dataset D561.**

| **Subject** | **Subtype** | **Defined*** | **T/Fs**** | **SCBD(%)** | **HD Q10** | **Fiebig Stage** | **Days post**  **Seroconversion** | **Risk***** | **HAART**  **Treat** | **Nseq** | **Sampling**  **Date** | **PMID** |
| --- | --- | --- | --- | --- | --- | --- | --- | --- | --- | --- | --- | --- |
| 62995 | B | Incident | 1 | 0.01 | 0 | I | - | SPD | Naïve | 27 | 1997-1-3 | 18490657 |
| 1054 | B | Incident | 1 | 0.03 | 0 | II | - | SPD | Naïve | 38 | 1997-11-30 | 18490657 |
| 1056 | B | Incident | 1 | 0.05 | 0 | II | - | SPD | Naïve | 46 | 1998-1-14 | 18490657 |
| 6240 | B | Incident | 1 | 0.02 | 0 | II | - | SPD | Naïve | 17 | 1995-11-22 | 18490657 |
| 6244 | B | Incident | 1 | 0.03 | 0 | II | - | SPD | Naïve | 11 | 1996-6-25 | 18490657 |
| 9010 | B | Incident | 1 | 0.04 | 0 | II | - | SPD | Naïve | 19 | 1997-11-25 | 18490657 |
| 9014 | B | Incident | 1 | 0.03 | 0 | II | - | SPD | Naïve | 13 | 1997-11-13 | 18490657 |
| 9015 | B | Incident | 1 | 0.02 | 0 | II | - | SPD | Naïve | 36 | 1997-12-27 | 18490657 |
| 9017 | B | Incident | 1 | 0.03 | 0 | II | - | SPD | Naïve | 26 | 1997-12-26 | 18490657 |
| 9020 | B | Incident | 1 | 0.02 | 0 | II | - | SPD | Naïve | 25 | 1998-6-16 | 18490657 |
| 9021 | B | Incident | 1 | 0.01 | 0 | II | - | SPD | Naïve | 34 | 1998-6-10 | 18490657 |
| 9023 | B | Incident | 1 | 0.02 | 0 | II | - | SPD | Naïve | 18 | 1998-7-7 | 18490657 |
| 9024 | B | Incident | 1 | 0.10 | 0 | II | - | SPD | Naïve | 23 | 1997-7-29 | 18490657 |
| 9025 | B | Incident | 1 | 0.06 | 0 | II | - | SPD | Naïve | 19 | 1998-8-16 | 18490657 |
| 9028 | B | Incident | 1 | 0.02 | 0 | II | - | SPD | Naïve | 23 | 1998-7-29 | 18490657 |
| 9075 | B | Incident | 1 | 0.03 | 0 | II | - | SPD | Naïve | 22 | 1996-9-13 | 18490657 |
| 9077 | B | Incident | 1 | 0.07 | 0 | II | - | SPD | Naïve | 24 | 1999-12-4 | 18490657 |
| 9079 | B | Incident | 1 | 0.05 | 0 | II | - | SPD | Naïve | 26 | 1999-12-11 | 18490657 |
| 61792 | B | Incident | 1 | 0.02 | 0 | II | - | SPD | Naïve | 18 | 1996-7-29 | 18490657 |
| 62130 | B | Incident | 1 | 0.03 | 0 | II | - | SPD | Naïve | 11 | 1996-9-3 | 18490657 |
| 62357 | B | Incident | 1 | 0.08 | 0 | II | - | SPD | Naïve | 14 | 1996-10-2 | 18490657 |
| 63054 | B | Incident | 1 | 0.10 | 0 | II | - | SPD | Naïve | 20 | 1997-1-19 | 18490657 |
| 63396 | B | Incident | 1 | 0.02 | 0 | II | - | SPD | Naïve | 21 | 1997-3-12 | 18490657 |

**Table S1**. **Detailed information of the dataset D561.**

| **Subject** | **Subtype** | **Defined*** | **T/Fs**** | **SCBD(%)** | **HD Q10** | **Fiebig Stage** | **Days post**  **Seroconversion** | **Risk***** | **HAART**  **Treat** | **Nseq** | **Sampling**  **Date** | **PMID** |
| --- | --- | --- | --- | --- | --- | --- | --- | --- | --- | --- | --- | --- |
| PRB926 | B | Incident | 1 | 0.03 | 0 | II | - | SPD | Naïve | 14 | 1994-2-19 | 18490657 |
| PRB956 | B | Incident | 1 | 0.03 | 0 | II | - | SPD | Naïve | 27 | 1997-8-19 | 18490657 |
| PRB959 | B | Incident | 1 | 0.00 | 0 | II | - | SPD | Naïve | 32 | 1999-11-17 | 18490657 |
| SC05 | B | Incident | 1 | 0.01 | 0 | II | - | SH | Naïve | 30 | 1993-6-28 | 18490657 |
| SC11 | B | Incident | 1 | 0.00 | 0 | II | - | SH | Naïve | 19 | 1993-10-11 | 18490657 |
| SC45 | B | Incident | 1 | 0.05 | 0 | II | - | SH | Naïve | 29 | 1995-1-18 | 18490657 |
| TRJO4551 | B | Incident | 1 | 0.14 | 0 | II | - | MSM | Naïve | 17 | 2001-10-10 | 18490657 |
| TT29P | B | Incident | 1 | 0.04 | 0 | II | - | SH | Naïve | 20 | 1998-3-20 | 18490657 |
| WEAU0575 | B | Incident | 1 | 0.03 | 0 | II | - | MSM | Naïve | 43 | 1990-5-30 | 18490657 |
| WITO4160 | B | Incident | 1 | 0.10 | 0 | II | - | SH | Naïve | 16 | 2000-8-4 | 18490657 |
| Z05 | B | Incident | 1 | 0.08 | 0 | II | - | SH | Naïve | 15 | 1998-9-21 | 18490657 |
| TT35P | B | Incident | 1 | 0.03 | 0 | II | - | SH | Naïve | 43 | 1999-1-26 | 18490657 |
| 63358 | B | Incident | 1 | 0.05 | 0 | II | - | SPD | Naïve | 27 | 1997-3-10 | 18490657 |
| 9029 | B | Incident | 1 | 0.07 | 0 | II | - | SPD | Naïve | 21 | 1998-9-10 | 18490657 |
| 12007 | B | Incident | 1 | 0.02 | 0 | II | - | SPD | Naïve | 25 | 1999-10-22 | 18490657 |
| SUMA0874 | B | Incident | 1 | 0.02 | 0 | II | - | MSM | Naïve | 35 | 1991-5-13 | 18490657 |
| SC22 | B | Incident | 1 | 0.04 | 0 | II | - | SH | Naïve | 38 | 1994-3-16 | 18490657 |
| 9030 | B | Incident | 1 | 0.04 | 0 | II | - | SPD | Naïve | 19 | 1998-10-2 | 18490657 |
| Z33 | B | Incident | 1 | 0.07 | 0 | II | - | SB | Naïve | 21 | 2002-10-12 | 18490657 |
| 6247 | B | Incident | 2 | 0.10 | 0 | II | - | SPD | Naïve | 31 | 1997-4-7 | 18490657 |
| TT31P | B | Incident | 2 | 0.13 | 0 | II | - | SH | Naïve | 67 | 1998-10-2 | 18490657 |
| Z31 | B | Incident | >1 | 0.26 | 0 | II | - | MSM | Naïve | 17 | 2001-12-9 | 18490657 |
| 63068 | B | Incident | 2 | 0.09 | 0 | II | - | SPD | Naïve | 20 | 1997-1-16 | 18490657 |

**Table S1**. **Detailed information of the dataset D561.**

| **Subject** | **Subtype** | **Defined*** | **T/Fs**** | **SCBD(%)** | **HD Q10** | **Fiebig Stage** | **Days post**  **Seroconversion** | **Risk***** | **HAART**  **Treat** | **Nseq** | **Sampling**  **Date** | **PMID** |
| --- | --- | --- | --- | --- | --- | --- | --- | --- | --- | --- | --- | --- |
| 62615 | B | Incident | 3 | 0.07 | 0 | II | - | SPD | Naïve | 28 | 1996-11-8 | 18490657 |
| SC33 | B | Incident | 2 | 0.06 | 0 | II | - | SH | Naïve | 27 | 1994-10-24 | 18490657 |
| BORI0637 | B | Incident | 5 | 0.06 | 0 | II | - | MSM | Naïve | 28 | 1990-8-23 | 18490657 |
| PRB957 | B | Incident | 4 | 0.09 | 0 | II | - | SPD | Naïve | 35 | 1999-4-28 | 18490657 |
| 12008 | B | Incident | 2 | 0.79 | 0 | II | - | SPD | Naïve | 30 | 1999-10-23 | 18490657 |
| 1006 | B | Incident | 1 | 0.05 | 0 | III | - | SPD | Naïve | 42 | 1997-6-5 | 18490657 |
| 9032 | B | Incident | 1 | 0.01 | 0 | III | - | SPD | Naïve | 39 | 1998-7-30 | 18490657 |
| 9033 | B | Incident | 1 | 0.02 | 0 | III | - | SPD | Naïve | 20 | 1998-3-29 | 18490657 |
| PRB931 | B | Incident | 1 | 0.04 | 0 | III | - | SPD | Naïve | 19 | 1995-9-11 | 18490657 |
| PRB958 | B | Incident | 1 | 0.03 | 0 | III | - | SPD | Naïve | 24 | 2000-2-10 | 18490657 |
| Z20 | B | Incident | 1 | 0.03 | 0 | III | - | MSM | Naïve | 26 | 2000-11-1 | 18490657 |
| Z34 | B | Incident | 1 | 0.13 | 0 | III | - | SB | Naïve | 18 | 2002-12-17 | 18490657 |
| 1018 | B | Incident | 1 | 0.04 | 0 | III | - | SPD | Naïve | 50 | 1997-6-20 | 18490657 |
| 1053 | B | Incident | 1 | 0.08 | 0 | III | - | SPD | Naïve | 58 | 1997-12-3 | 18490657 |
| 6248 | B | Incident | 1 | 0.12 | 0 | III | - | SPD | Naïve | 20 | 1997-3-9 | 18490657 |
| 1001 | B | Incident | 1 | 0.05 | 0 | III | - | SPD | Naïve | 62 | 1997-2-12 | 18490657 |
| 9022 | B | Incident | 1 | 0.05 | 0 | III | - | SPD | Naïve | 23 | 1997-8-31 | 18490657 |
| 1059 | B | Incident | 1 | 0.13 | 0 | III | - | SPD | Naïve | 39 | 1998-3-26 | 18490657 |
| 1012 | B | Incident | 1 | 0.03 | 0 | III | - | SPD | Naïve | 43 | 1997-4-7 | 18490657 |
| 700010058 | B | Incident | 1 | 0.07 | 0 | III | - | - | Naïve | 46 | 2006-8-31 | 18490657 |
| 9026 | B | Incident | 2 | 0.30 | 0 | III | - | SPD | Naïve | 15 | 1998-9-3 | 18490657 |
| 9076 | B | Incident | 2 | 0.30 | 0 | III | - | SPD | Naïve | 32 | 1997-4-28 | 18490657 |
| 1051 | B | Incident | 4 | 0.94 | 0 | III | - | SPD | Naïve | 50 | 1997-10-17 | 18490657 |

**Table S1**. **Detailed information of the dataset D561.**

| **Subject** | **Subtype** | **Defined*** | **T/Fs**** | **SCBD(%)** | **HD Q10** | **Fiebig Stage** | **Days post**  **Seroconversion** | **Risk***** | **HAART**  **Treat** | **Nseq** | **Sampling**  **Date** | **PMID** |
| --- | --- | --- | --- | --- | --- | --- | --- | --- | --- | --- | --- | --- |
| SC20 | B | Incident | 1 | 0.04 | 0 | IV | - | SH | Naïve | 42 | 1994-2-17 | 18490657 |
| Z32 | B | Incident | 1 | 0.04 | 0 | IV | - | MSM | Naïve | 10 | 2002-2-9 | 18490657 |
| 9031 | B | Incident | 1 | 0.02 | 0 | IV | - | SPD | Naïve | 20 | 1998-2-16 | 18490657 |
| 1058 | B | Incident | 1 | 0.10 | 0 | IV | - | SPD | Naïve | 45 | 1998-3-18 | 18490657 |
| SC31 | B | Incident | 1 | 0.09 | 0 | IV | - | SH | Naïve | 36 | 1994-9-15 | 18490657 |
| TT27P | B | Incident | 3 | 0.86 | 0 | IV | - | SH | Naïve | 38 | 1998-1-19 | 18490657 |
| Z35 | B | Incident | 2 | 0.12 | 0 | IV | - | MSM | Naïve | 21 | 2003-1-15 | 18490657 |
| SC42 | B | Incident | >3 | 0.67 | 1 | IV | - | SH | Naïve | 25 | 1995-1-18 | 18490657 |
| 9019 | B | Incident | 1 | 0.11 | 0 | V | - | SPD | Naïve | 18 | 1997-12-28 | 18490657 |
| REJO4541 | B | Incident | 1 | 0.04 | 0 | V | - | SH | Naïve | 21 | 2001-9-28 | 18490657 |
| THRO4156 | B | Incident | 1 | 0.05 | 0 | V | - | MSM | Naïve | 27 | 2000-8-1 | 18490657 |
| TT28P | B | Incident | 1 | 0.01 | 0 | V | - | SH | Naïve | 28 | 1998-1-20 | 18490657 |
| TT34P | B | Incident | 1 | 0.04 | 0 | V | - | SH | Naïve | 29 | 1998-1-13 | 18490657 |
| Z02 | B | Incident | 1 | 0.07 | 0 | V | - | SH | Naïve | 21 | 1998-5-27 | 18490657 |
| Z13 | B | Incident | 1 | 0.11 | 0 | V | - | SH | Naïve | 31 | 1999-5-17 | 18490657 |
| Z23 | B | Incident | 1 | 0.08 | 0 | V | - | SB | Naïve | 15 | 2001-2-1 | 18490657 |
| Z27 | B | Incident | 1 | 0.07 | 0 | V | - | SH | Naïve | 25 | 2001-6-20 | 18490657 |
| RHPA4259 | B | Incident | 1 | 0.06 | 0 | V | - | SH | Naïve | 31 | 2000-12-5 | 18490657 |
| 63215 | B | Incident | 1 | 0.04 | 0 | V | - | SPD | Naïve | 19 | 1997-2-12 | 18490657 |
| SC51 | B | Incident | 1 | 0.04 | 0 | V | - | SH | Naïve | 33 | 1996-1-15 | 18490657 |
| 700010040 | B | Incident | 1 | 0.03 | 0 | V | - | MSM | Naïve | 29 | 2006-7-27 | 18490657 |
| 700010077 | B | Incident | 1 | 0.19 | 0 | V | - | - | Naïve | 51 | 2006-9-8 | 18490657 |
| MEMI4948 | B | Incident | 1 | 0.12 | 0 | V | - | MSM | Naïve | 32 | 2003-1-28 | 18490657 |

**Table S1**. **Detailed information of the dataset D561.**

| **Subject** | **Subtype** | **Defined*** | **T/Fs**** | **SCBD(%)** | **HD Q10** | **Fiebig Stage** | **Days post**  **Seroconversion** | **Risk***** | **HAART**  **Treat** | **Nseq** | **Sampling**  **Date** | **PMID** |
| --- | --- | --- | --- | --- | --- | --- | --- | --- | --- | --- | --- | --- |
| 700010019 | B | Incident | >3 | 0.21 | 0 | V | - | IDU | Naïve | 33 | 2006-6-20 | 18490657 |
| Z18 | B | Incident | 3 | 0.04 | 0 | V | - | MSM | Naïve | 34 | 2000-6-30 | 18490657 |
| CAAN5342 | B | Incident | >2 | 0.79 | 0 | V | - | MSM | Naïve | 40 | 2004-4-13 | 18490657 |
| Z30 | B | Incident | 2 | 0.36 | 0 | V | - | SH | Naïve | 30 | 2001-11-5 | 18490657 |
| Z16 | B | Incident | 5 | 0.04 | 0 | V | - | MSM | Naïve | 19 | 1999-8-30 | 18490657 |
| Z03 | B | Incident | 3 | 0.27 | 0 | V | - | MSM | Naïve | 22 | 1998-8-6 | 18490657 |
| 701010016 | B | Incident | 2 | 0.32 | 0 | V | - | - | Naïve | 20 | 2006-7-24 | 18490657 |
| Z29 | B | Incident | >3 | 0.03 | 0 | V | - | SB | Naïve | 16 | 2001-8-13 | 18490657 |
| Z36 | B | Incident | 1 | 0.02 | 0 | VI | - | - | Naïve | 17 | 2003-1-29 | 18490657 |
| Z10 | B | Incident | >2 | 0.65 | 3 | VI | - | MSM | Naïve | 17 | 1999-3-24 | 18490657 |
| 1058 | B | Incident | 1 | 0.01 | 0 | I | - | SPD | Naïve | 36 | 1998-3-8 | 18490657 |
| 1058 | B | Incident | 1 | 0.08 | 0 | II | - | SPD | Naïve | 18 | 1998-3-11 | 18490657 |
| SUMA0874 | B | Incident | 1 | 0.04 | 0 | II | - | MSM | Naïve | 26 | 1991-5-12 | 18490657 |
| SUMA0874 | B | Incident | 1 | 0.06 | 0 | III | - | MSM | Naïve | 31 | 1991-5-21 | 18490657 |
| SUMA0874 | B | Incident | 1 | 0.08 | 0 | IV | - | MSM | Naïve | 24 | 1991-5-28 | 18490657 |
| SUMA0874 | B | Incident | 1 | 0.01 | 0 | V | - | MSM | Naïve | 28 | 1991-6-11 | 18490657 |
| WEAU0575 | B | Incident | 1 | 0.03 | 0 | IV | - | MSM | Naïve | 44 | 1990-6-7 | 18490657 |
| WEAU0575 | B | Incident | 1 | 0.08 | 0 | IV | - | MSM | Naïve | 58 | 1990-6-14 | 18490657 |
| WEAU0575 | B | Incident | 2 | 0.16 | 0 | V | - | MSM | Naïve | 30 | 1990-6-28 | 18490657 |
| WEAU0575 | B | Incident | >2 | 0.57 | 1 | VI | - | MSM | Naïve | 27 | 1990-12-13 | 18490657 |
| 1051 | B | Incident | 4 | 0.01 | 0 | I | - | SPD | Naïve | 27 | 1997-10-7 | 18490657 |
| 1059 | B | Incident | 1 | 0.08 | 0 | II | - | SPD | Naïve | 46 | 1998-3-19 | 18490657 |
| 1059 | B | Incident | 1 | 0.04 | 0 | II | - | SPD | Naïve | 51 | 1998-3-23 | 18490657 |

**Table S1**. **Detailed information of the dataset D561.**

| **Subject** | **Subtype** | **Defined*** | **T/Fs**** | **SCBD(%)** | **HD Q10** | **Fiebig Stage** | **Days post**  **Seroconversion** | **Risk***** | **HAART**  **Treat** | **Nseq** | **Sampling**  **Date** | **PMID** |
| --- | --- | --- | --- | --- | --- | --- | --- | --- | --- | --- | --- | --- |
| 1056 | B | Incident | 1 | 0.00 | 0 | I | - | SPD | Naïve | 22 | 1998-1-7 | 18490657 |
| 1056 | B | Incident | 1 | 0.03 | 0 | II | - | SPD | Naïve | 37 | 1998-1-10 | 18490657 |
| 6247 | B | Incident | 2 | 0.09 | 0 | I | - | SPD | Naïve | 25 | 1997-4-5 | 18490657 |
| 700010040 | B | Incident | 1 | 0.08 | 0 | II | - | MSM | Naïve | 45 | 2006-7-11 | 18490657 |
| 700010077 | B | Incident | 1 | 0.07 | 0 | II | - | MSM | Naïve | 22 | 2006-8-25 | 18490657 |
| TT31P | B | Incident | 2 | 0.06 | 0 | IV | - | SH | Naïve | 6 | 1998-10-13 | 18490657 |
| CRPE4571 | B | Chronic | - | 2.43 | 7 | - | >730 | SH | Naïve | 16 | 2001-11-30 | 18490657 |
| FERI5029 | B | Chronic | - | 2.47 | 11 | - | >545 | MSM | Naïve | 14 | 2003-9-17 | 18490657 |
| FOJO4081 | B | Chronic | - | 1.89 | 6 | - | >5,715 | MSM | Naïve | 31 | 2002-6-3 | 18490657 |
| HEMA4284 | B | Chronic | - | 2.00 | 4 | - | >545 | MSM | Naïve | 46 | 2002-10-2 | 18490657 |
| JOTO5278 | B | Chronic | - | 3.11 | 4 | - | >545 | SH | Naïve | 21 | 2004-2-13 | 18490657 |
| LAHA4867 | B | Chronic | - | 1.76 | 1.5 | - | >730 | MSM | Naïve | 21 | 2004-10-25 | 18490657 |
| MCRO3633 | B | Chronic | - | 2.00 | 1 | - | >2,735 | SH | Naïve | 21 | 2006-1-24 | 18490657 |
| OLLA4645 | B | Chronic | - | 1.62 | 3 | - | >740 | SH | Naïve | 27 | 2002-2-22 | 18490657 |
| SAMI4303 | B | Chronic | - | 3.83 | 6 | - | >1,430 | MSM | Naïve | 39 | 2004-8-5 | 18490657 |
| SHKE4761 | B | Chronic | - | 1.75 | 2 | - | >425 | MSM | Naïve | 24 | 2002-8-6 | 18490657 |
| SMRE4166 | B | Chronic | - | 1.30 | 4 | - | >485 | SH | Naïve | 24 | 2001-11-16 | 18490657 |
| TALA4022 | B | Chronic | - | 4.53 | 9 | - | >2,525 | MSM | Naïve | 37 | 2003-12-9 | 18490657 |
| WICU4248 | B | Chronic | - | 1.90 | 5 | - | >2,160 | MSM | Naïve | 38 | 2005-10-6 | 18490657 |
| YOMI4024 | B | Chronic | - | 1.89 | 5 | - | >2,220 | MSM | Naïve | 23 | 2006-2-15 | 18490657 |
| UNC3405 | B | Chronic | - | 3.03 | 11 | - | >2,890 | MSM | Naïve | 16 | 2004-3-10 | 18490657 |
| UNC5057 | B | Chronic | - | 3.10 | 6 | - | >1,765 | SH | Naïve | 14 | 2005-6-8 | 18490657 |
| UNC5417 | B | Chronic | - | 3.02 | 9 | - | >1,155 | SH | Naïve | 11 | 2004-7-26 | 18490657 |

**Table S1**. **Detailed information of the dataset D561.**

| **Subject** | **Subtype** | **Defined*** | **T/Fs**** | **SCBD(%)** | **HD Q10** | **Fiebig Stage** | **Days post**  **Seroconversion** | **Risk***** | **HAART**  **Treat** | **Nseq** | **Sampling**  **Date** | **PMID** |
| --- | --- | --- | --- | --- | --- | --- | --- | --- | --- | --- | --- | --- |
| UNC5479 | B | Chronic | - | 2.35 | 4 | - | >1,670 | SH | Naïve | 21 | 2005-8-2 | 18490657 |
| UNC5539 | B | Chronic | - | 2.58 | 8 | - | >1,855 | MSM | Naïve | 15 | 2004-10-28 | 18490657 |
| UNC5769 | B | Chronic | - | 2.41 | 1 | - | >950 | MSM | Naïve | 16 | 2004-4-2 | 18490657 |
| UNC5791 | B | Chronic | - | 4.70 | 2 | - | >1,580 | SH | Naïve | 9 | 2005-9-14 | 18490657 |
| UNC7092 | B | Chronic | - | 2.28 | 2 | - | >5,475 | SH | Naïve | 11 | 2004-12-30 | 18490657 |
| UNC2009 | B | Chronic | - | 2.87 | 3 | - | >1,430 | MSM | Naïve | 11 | 2004-4-9 | 18490657 |
| UNC4295 | B | Chronic | - | 2.88 | 3 | - | >2,705 | MSM | Naïve | 22 | 2004-9-1 | 18490657 |
| UNC4484 | B | Chronic | - | 3.43 | 7 | - | >2,250 | SH | Naïve | 19 | 2004-5-4 | 18490657 |
| UNC4911 | B | Chronic | - | 3.88 | 8 | - | >1,885 | SH | Naïve | 19 | 2004-3-23 | 18490657 |
| UNC5283 | B | Chronic | - | 2.78 | 11 | - | >1,520 | SH | Naïve | 23 | 2004-8-16 | 18490657 |
| UNC5548 | B | Chronic | - | 3.11 | 7 | - | >1,305 | SH | Naïve | 21 | 2004-4-27 | 18490657 |
| UNC5734 | B | Chronic | - | 1.78 | 1 | - | >1,370 | SH,IDU | Naïve | 12 | 2004-2-2 | 18490657 |
| UNC5799 | B | Chronic | - | 3.17 | 6 | - | >4,015 | MSM | Naïve | 24 | 2004-4-19 | 18490657 |
| UNC6064 | B | Chronic | - | 1.29 | 1 | - | >1,490 | MSM | Naïve | 15 | 2004-3-4 | 18490657 |
| SC02 | B | Chronic | - | 1.94 | 6 | - | >1,095 | SH | Naïve | 35 | 1996-4-15 | 18490657 |
| SC03 | B | Chronic | - | 3.39 | 5 | - | >2,525 | SH | Naïve | 24 | 2000-5-24 | 18490657 |
| SC05 | B | Chronic | - | 2.73 | 1 | - | >1,975 | SH | Naïve | 11 | 1998-12-9 | 18490657 |
| SC13 | B | Chronic | - | 2.13 | 4 | - | >970 | SH | Naïve | 20 | 1996-2-28 | 18490657 |
| SC24 | B | Chronic | - | 2.18 | 2 | - | >1,395 | SH | Naïve | 24 | 1998-5-26 | 18490657 |
| SC25 | B | Chronic | - | 1.48 | 3 | - | >1,000 | SH | Naïve | 17 | 1997-4-9 | 18490657 |
| SC51 | B | Chronic | - | 2.82 | 5 | - | >545 | SH | Naïve | 19 | 1997-7-7 | 18490657 |
| TT103 | B | Chronic | - | 1.41 | 2 | - | >455 | SH | Naïve | 19 | 2001-4-10 | 18490657 |
| TT112P | B | Chronic | - | 1.78 | 3 | - | >435 | SH | Naïve | 15 | 2001-6-6 | 18490657 |

**Table S1**. **Detailed information of the dataset D561.**

| **Subject** | **Subtype** | **Defined*** | **T/Fs**** | **SCBD(%)** | **HD Q10** | **Fiebig Stage** | **Days post**  **Seroconversion** | **Risk***** | **HAART**  **Treat** | **Nseq** | **Sampling**  **Date** | **PMID** |
| --- | --- | --- | --- | --- | --- | --- | --- | --- | --- | --- | --- | --- |
| TT113P | B | Chronic | - | 1.65 | 5 | - | >365 | SH | Naïve | 23 | 2001-4-21 | 18490657 |
| TT114P | B | Chronic | - | 1.80 | 5 | - | >365 | SH | Naïve | 23 | 2001-5-15 | 18490657 |
| TT31P | B | Chronic | - | 1.26 | 5 | - | >730 | SH | Naïve | 14 | 2000-10-9 | 18490657 |
| Z91 | B | Indident | 1 | 0.13 | 0 | V | - | MSM | Naïve | 17 | 2006 | 21980282 |
| UW1218 | B | Chronic | - | 1.01 | 2 | - | >730 | MSM | Naïve | 29 | 2004 | 21980282 |
| UW1249 | B | Chronic | - | 1.82 | 4 | - | >730 | MSM | Naïve | 37 | 2004 | 21980282 |
| UW1262 | B | Chronic | - | 2.48 | 8 | - | >730 | IDU | Naïve | 37 | 2004 | 21980282 |
| UW1330 | B | Chronic | - | 5.08 | 10 | - | >730 | IDU | Naïve | 30 | 2004 | 21980282 |
| UW1352 | B | Chronic | - | 1.70 | 2 | - | >730 | MSM | Naïve | 56 | 2004 | 21980282 |
| UW1423 | B | Chronic | - | 2.11 | 9 | - | >730 | MSM | Naïve | 20 | 2005 | 21980282 |
| UW1444 | B | Chronic | - | 4.38 | 5 | - | >730 | MSM | Naïve | 24 | 2005 | 21980282 |
| UW1446 | B | Chronic | - | 1.51 | 6 | - | >730 | MSM | Naïve | 31 | 2005 | 21980282 |
| UW1451 | B | Chronic | - | 3.54 | 6 | - | >730 | MSM | Naïve | 43 | 2005 | 21980282 |
| UW1470 | B | Chronic | - | 3.02 | 10 | - | >730 | MSM | Naïve | 27 | 2005 | 21980282 |
| UW1508 | B | Chronic | - | 3.12 | 5 | - | >730 | MSM | Naïve | 28 | 2005 | 21980282 |
| UW1586 | B | Chronic | - | 2.69 | 4 | - | >730 | MSM | Naïve | 25 | 2005 | 21980282 |
| UW1588 | B | Chronic | - | 5.42 | 8 | - | >730 | MSM | Naïve | 30 | 2005 | 21980282 |
| UW1599 | B | Chronic | - | 4.91 | 4 | - | >730 | IDU | Naïve | 25 | 2005 | 21980282 |
| UW1624 | B | Chronic | - | 3.08 | 4 | - | >730 | MSM | Naïve | 31 | 2005 | 21980282 |
| UW1631 | B | Chronic | - | 1.13 | 1 | - | >730 | SH | Naïve | 20 | 2005 | 21980282 |
| UW1632 | B | Chronic | - | 1.20 | 3 | - | >730 | MSM | Naïve | 32 | 2005 | 21980282 |
| UW1711 | B | Chronic | - | 1.37 | 4 | - | >730 | IDU | Naïve | 22 | 2006 | 21980282 |
| UW1791 | B | Chronic | - | 3.10 | 5 | - | >730 | SH | Naïve | 34 | 2006 | 21980282 |

**Table S1**. **Detailed information of the dataset D561.**

| **Subject** | **Subtype** | **Defined*** | **T/Fs**** | **SCBD(%)** | **HD Q10** | **Fiebig Stage** | **Days post**  **Seroconversion** | **Risk***** | **HAART**  **Treat** | **Nseq** | **Sampling**  **Date** | **PMID** |
| --- | --- | --- | --- | --- | --- | --- | --- | --- | --- | --- | --- | --- |
| UW1794 | B | Chronic | - | 1.71 | 1 | - | >730 | IDU | Naïve | 22 | 2006 | 21980282 |
| 700010224 | B | Incident | >1 | 0.35 | 0 | V | - | - | Naïve | 48 | 2007 | 21980282 |
| 701010043 | B | Incident | 1 | 0.06 | 0 | II | - | NR | Naïve | 24 | 2006 | 21980282 |
| 701010092 | B | Incident | >1 | 0.93 | 0 | V | - | - | Naïve | 36 | 2007 | 21980282 |
| AVDA2874 | B | Chronic | - | 4.36 | 2 | - | >730 | MSM | Naïve | 20 | 2007 | 21980282 |
| BEKA5842 | B | Chronic | - | 2.29 | 6.5 | - | >730 | SH | Naïve | 16 | 2007 | 21980282 |
| BRJO4843 | B | Chronic | - | 1.83 | 4 | - | >730 | MSM | Naïve | 29 | 2007 | 21980282 |
| COCE6096 | B | Chronic | - | 2.98 | 5 | - | >730 | MSM | Naïve | 23 | 2007 | 21980282 |
| DIGA3757 | B | Chronic | - | 2.15 | 4 | - | >730 | MSM | Naïve | 22 | 2004 | 21980282 |
| EABE4469 | B | Chronic | - | 4.93 | 9 | - | >730 | MSM | Naïve | 31 | 2002 | 21980282 |
| FASH1057 | B | Incident | 1 | 0.02 | 0 | II | - | SH | Naïve | 31 | 1991 | 21980282 |
| GETO5098 | B | Chronic | - | 1.63 | 5 | - | >730 | MSM | Naïve | 27 | 2006 | 21980282 |
| HALA6323 | B | Chronic | - | 2.83 | 9 | - | >730 | MSM | Naïve | 32 | 2007 | 21980282 |
| JACH1853 | B | Chronic | - | 2.71 | 6 | - | >730 | SH | Naïve | 25 | 2004 | 21980282 |
| JOSA5789 | B | Chronic | - | 1.85 | 4 | - | >730 | SH | Naïve | 28 | 2007 | 21980282 |
| MCBR4209 | B | Chronic | - | 1.72 | 2 | - | >730 | IDU | Naïve | 25 | 2007 | 21980282 |
| MCST4474 | B | Chronic | - | 3.49 | 3 | - | >730 | MSM | Naïve | 25 | 2007 | 21980282 |
| MEJA5586 | B | Chronic | - | 2.17 | 6 | - | >730 | MSM | Naïve | 25 | 2007 | 21980282 |
| RHGA1581 | B | Chronic | - | 3.97 | 4 | - | >730 | MSM | Naïve | 31 | 2003 | 21980282 |
| RHMI4089 | B | Chronic | - | 1.77 | 1 | - | >730 | MSM | Naïve | 31 | 2005 | 21980282 |
| ROCH4447 | B | Chronic | - | 1.59 | 2 | - | >730 | MSM | Naïve | 33 | 2007 | 21980282 |
| ROST4216 | B | Chronic | - | 2.00 | 4 | - | >730 | MSM | Naïve | 33 | 2005 | 21980282 |
| SADO6038 | B | Chronic | - | 2.50 | 9 | - | >730 | SH | Naïve | 30 | 2007 | 21980282 |

**Table S1**. **Detailed information of the dataset D561.**

| **Subject** | **Subtype** | **Defined*** | **T/Fs**** | **SCBD(%)** | **HD Q10** | **Fiebig Stage** | **Days post**  **Seroconversion** | **Risk***** | **HAART**  **Treat** | **Nseq** | **Sampling**  **Date** | **PMID** |
| --- | --- | --- | --- | --- | --- | --- | --- | --- | --- | --- | --- | --- |
| SC13 | B | Incident | 1 | 0.05 | 0 | II/III | - | SH | Naïve | 23 | 1996 | 21980282 |
| SC24 | B | Incident | 1 | 0.03 | 0 | II/III | - | SH | Naïve | 24 | 1998 | 21980282 |
| SC46 | B | Chronic | - | 2.62 | 4 | - | >730 | SH | Naïve | 22 | 2000 | 21980282 |
| SC50 | B | Incident | >1 | 0.35 | 0 | II/III | - | SH | Naïve | 35 | 1995 | 21980282 |
| SPFE4120 | B | Chronic | - | 2.11 | 0 | - | >730 | SH | Naïve | 25 | 2007 | 21980282 |
| STCO5453 | B | Chronic | - | 1.55 | 2 | - | >730 | MSM | Naïve | 27 | 2005 | 21980282 |
| TT106 | B | Chronic | - | 2.95 | 6 | - | >730 | SH | Naïve | 24 | 2001 | 21980282 |
| WARO5662 | B | Chronic | - | 2.44 | 1 | - | >730 | SH | Naïve | 34 | 2007 | 21980282 |
| Z62 | B | Incident | 1 | 0.05 | 0 | VI | - | MSM | Naïve | 22 | 2005 | 21980282 |
| Z64 | B | Incident | >1 | 1.55 | 4 | IV | - | SH | Naïve | 20 | 2005 | 21980282 |
| Z71 | B | Incident | 1 | 0.16 | 0 | VI | - | SH | Naïve | 14 | 2005 | 21980282 |
| Z74 | B | Incident | >1 | 0.05 | 0 | VI | - | - | Naïve | 18 | 2005 | 21980282 |
| Z75 | B | Incident | 1 | 0.05 | 0 | V | - | MSM | Naïve | 22 | 2005 | 21980282 |
| Z78 | B | Incident | 1 | 0.14 | 0 | V | - | MSM | Naïve | 16 | 2005 | 21980282 |
| Z85 | B | Incident | 1 | 0.09 | 0 | VI | - | MSM | Naïve | 17 | 2006 | 21980282 |
| Z86 | B | Incident | >1 | 0.33 | 0 | VI | - | MSM | Naïve | 20 | 2006 | 21980282 |
| Z92 | B | Incident | 1 | 0.31 | 0 | V | - | MSM | Naïve | 17 | 2006 | 21980282 |
| Z93 | B | Incident | 1 | 0.34 | 1 | VI | - | MSM | Naïve | 19 | 2006 | 21980282 |
| Z94 | B | Incident | >1 | 0.09 | 0 | VI | - | MSM | Naïve | 14 | 2006 | 21980282 |
| Z95 | B | Incident | >1 | 0.70 | 0 | V | - | SH | Naïve | 19 | 2006 | 21980282 |
| CAP45 | C | Incident | 1 | 0.00 | 0 | I/II | - | SH | Naïve | 16 | 2005-4-20 | 19193811 |
| CAP129 | C | Incident | 1 | 0.06 | 0 | IV | - | SH | Naïve | 19 | 2006-6-13 | 19193811 |
| CAP174 | C | Incident | 1 | 0.07 | 0 | V | - | SH | Naïve | 21 | 2005-9-22 | 19193811 |

**Table S1**. **Detailed information of the dataset D561.**

| **Subject** | **Subtype** | **Defined*** | **T/Fs**** | **SCBD(%)** | **HD Q10** | **Fiebig Stage** | **Days post**  **Seroconversion** | **Risk***** | **HAART**  **Treat** | **Nseq** | **Sampling**  **Date** | **PMID** |
| --- | --- | --- | --- | --- | --- | --- | --- | --- | --- | --- | --- | --- |
| CAP177 | C | Incident | 1 | 0.02 | 0 | I/II | - | SH | Naïve | 20 | 2006-3-7 | 19193811 |
| CAP188 | C | Incident | 1 | 0.05 | 0 | I/II | - | SH | Naïve | 22 | 2007-1-25 | 19193811 |
| CAP200 | C | Incident | 1 | 0.11 | 0 | IV | - | SH | Naïve | 18 | 2005-10-11 | 19193811 |
| CAP206 | C | Incident | 1 | 0.04 | 0 | V | - | SH | Naïve | 21 | 2005-6-15 | 19193811 |
| CAP210 | C | Incident | 1 | 0.02 | 0 | I/II | - | SH | Naïve | 21 | 2005-5-3 | 19193811 |
| CAP217 | C | Incident | 1 | 0.04 | 0 | IV | - | SH | Naïve | 20 | 2005-12-2 | 19193811 |
| CAP220 | C | Incident | 1 | 0.12 | 0 | V | - | SH | Naïve | 15 | 2007-2-15 | 19193811 |
| CAP221 | C | Incident | 1 | 0.06 | 0 | I/II | - | SH | Naïve | 21 | 2006-3-2 | 19193811 |
| CAP237 | C | Incident | 1 | 0.05 | 0 | III | - | SH | Naïve | 22 | 2007-3-6 | 19193811 |
| CAP239 | C | Incident | 1 | 0.13 | 0 | V | - | SH | Naïve | 24 | 2005-8-10 | 19193811 |
| 089 | C | Incident | 1 | 0.07 | 0 | V | - | SH | Naïve | 22 | 2003-4-10 | 19193811 |
| 0334 | C | Incident | 1 | 0.02 | 0 | I/II | - | SH | Naïve | 22 | 2003-6-30 | 19193811 |
| 0393 | C | Incident | 1 | 0.04 | 0 | IV | - | SH | Naïve | 22 | 2003-7-17 | 19193811 |
| 0626 | C | Incident | 1 | 0.05 | 0 | IV | - | SH | Naïve | 24 | 2003-9-16 | 19193811 |
| 0665 | C | Incident | 1 | 0.04 | 0 | IV | - | SH | Naïve | 20 | 2003-9-24 | 19193811 |
| 0682 | C | Incident | 1 | 0.00 | 0 | I/II | - | SH | Naïve | 22 | 2003-9-29 | 19193811 |
| 0985 | C | Incident | 1 | 0.05 | 0 | I/II | - | SH | Naïve | 23 | 2004-2-11 | 19193811 |
| 1172 | C | Incident | 1 | 0.06 | 0 | I/II | - | SH | Naïve | 20 | 2004-5-13 | 19193811 |
| 2052 | C | Incident | 1 | 0.00 | 0 | I/II | - | SH | Naïve | 23 | 2006-3-24 | 19193811 |
| 2060 | C | Incident | 1 | 0.02 | 0 | I/II | - | SH | Naïve | 22 | 2005-5-4 | 19193811 |
| 703010131 | C | Incident | 1 | 0.05 | 0 | III | - | SH | Naïve | 22 | 2007-3-15 | 19193811 |
| 703010193 | C | Incident | 1 | 0.08 | 0 | V | - | SH | Naïve | 24 | 2007-5-8 | 19193811 |
| 703010217 | C | Incident | 1 | 0.08 | 0 | V/VI | - | SH | Naïve | 25 | 2007-1-4 | 19193811 |

**Table S1**. **Detailed information of the dataset D561.**

| **Subject** | **Subtype** | **Defined*** | **T/Fs**** | **SCBD(%)** | **HD Q10** | **Fiebig Stage** | **Days post**  **Seroconversion** | **Risk***** | **HAART**  **Treat** | **Nseq** | **Sampling**  **Date** | **PMID** |
| --- | --- | --- | --- | --- | --- | --- | --- | --- | --- | --- | --- | --- |
| 704010042 | C | Incident | 1 | 0.07 | 0 | V | - | SH | Naïve | 42 | 2007-2-2 | 19193811 |
| 704010083 | C | Incident | 1 | 0.05 | 0 | III | - | SH | Naïve | 24 | 2007-2-19 | 19193811 |
| 704809221 | C | Incident | 1 | 0.09 | 0 | I/II | - | SH | Naïve | 28 | 2007-8-13 | 19193811 |
| 706010164 | C | Incident | 1 | 0.42 | 0 | IV | - | SH | Naïve | 19 | 2007-8-16 | 19193811 |
| CAP40 | C | Incident | 1 | 0.12 | 0 | VI | - | SH | Naïve | 22 | 2006-4-25 | 19193811 |
| CAP63 | C | Incident | 1 | 0.06 | 0 | III | - | SH | Naïve | 19 | 2005-1-6 | 19193811 |
| CAP84 | C | Incident | 1 | 0.05 | 0 | V | - | SH | Naïve | 22 | 2005-2-16 | 19193811 |
| CAP85 | C | Incident | 1 | 0.25 | 0 | V | - | SH | Naïve | 21 | 2005-6-22 | 19193811 |
| CAP225 | C | Incident | 1 | 0.04 | 0 | III | - | SH | Naïve | 20 | 2005-10-25 | 19193811 |
| 1086 | C | Incident | 1 | 0.06 | 0 | I/II | - | SH | Naïve | 24 | 2004-3-22 | 19193811 |
| 2010 | C | Incident | 1 | 0.05 | 0 | IV | - | SH | Naïve | 23 | 2005-1-19 | 19193811 |
| 704810053 | C | Incident | 1 | 0.04 | 0 | V | - | SH | Naïve | 20 | 2007-9-11 | 19193811 |
| 1176 | C | Incident | 3 | 0.07 | 0 | I/II | - | SH | Naïve | 21 | 2004-5-14 | 19193811 |
| 1373 | C | Incident | 1 | 0.00 | 0 | I/II | - | SH | Naïve | 22 | 2004-8-27 | 19193811 |
| 1394 | C | Incident | 1 | 0.02 | 0 | I/II | - | SH | Naïve | 20 | 2004-9-7 | 19193811 |
| 2103 | C | Incident | 1 | 0.02 | 0 | I/II | - | SH | Naïve | 20 | 2005-7-21 | 19193811 |
| 703010159 | C | Incident | 1 | 0.02 | 0 | II | - | SH | Naïve | 20 | 2007-3-23 | 19193811 |
| CAP8 | C | Incident | 1 | 0.07 | 0 | V | - | SH | Naïve | 19 | 2005-5-11 | 19193811 |
| CAP269 | C | Incident | 1 | 0.23 | 0 | VI | - | SH | Naïve | 17 | 2006-9-27 | 19193811 |
| 0595 | C | Incident | 1 | 0.01 | 0 | IV | - | SH | Naïve | 28 | 2003-9-11 | 19193811 |
| 703010054 | C | Incident | 1 | 0.19 | 0 | V | - | SH | Naïve | 27 | 2007-1-19 | 19193811 |
| 704010017 | C | Incident | 1 | 0.03 | 0 | VI | - | SH | Naïve | 25 | 2006-12-5 | 19193811 |
| 704010056 | C | Incident | 1 | 0.17 | 0 | VI | - | SH | Naïve | 21 | 2007-2-13 | 19193811 |

**Table S1**. **Detailed information of the dataset D561.**

| **Subject** | **Subtype** | **Defined*** | **T/Fs**** | **SCBD(%)** | **HD Q10** | **Fiebig Stage** | **Days post**  **Seroconversion** | **Risk***** | **HAART**  **Treat** | **Nseq** | **Sampling**  **Date** | **PMID** |
| --- | --- | --- | --- | --- | --- | --- | --- | --- | --- | --- | --- | --- |
| 704010069 | C | Incident | 1 | 0.13 | 0 | VI | - | SH | Naïve | 23 | 2007-2-14 | 19193811 |
| 705010015 | C | Incident | 1 | 0.03 | 0 | V | - | SH | Naïve | 23 | 2007-1-12 | 19193811 |
| 705010110 | C | Incident | 1 | 0.15 | 0 | VI | - | SH | Naïve | 20 | 2007-3-26 | 19193811 |
| 706010018 | C | Incident | 1 | 0.37 | 1 | VI | - | SH | Naïve | 23 | 2007-6-4 | 19193811 |
| 705010026 | C | Incident | 1 | 0.04 | 0 | V | - | SH | Naïve | 23 | 2007-2-1 | 19193811 |
| 705010078 | C | Incident | 1 | 0.05 | 0 | V | - | SH | Naïve | 26 | 2007-2-27 | 19193811 |
| CAP37 | C | Incident | 3 | 0.05 | 0 | IV | - | SH | Naïve | 20 | 2006-5-11 | 19193811 |
| CAP69 | C | Incident | 5 | 0.39 | 0 | I/II | - | SH | Naïve | 20 | 2006-1-31 | 19193811 |
| CAP136 | C | Incident | 2 | 0.39 | 0 | V | - | SH | Naïve | 16 | 2006-6-29 | 19193811 |
| CAP222 | C | Incident | 3 | 0.96 | 0 | I/II | - | SH | Naïve | 21 | 2006-4-5 | 19193811 |
| CAP224 | C | Incident | 2 | 0.31 | 0 | V | - | SH | Naïve | 19 | 2006-11-15 | 19193811 |
| CAP260 | C | Incident | 2 | 0.14 | 0 | V | - | SH | Naïve | 18 | 2006-2-8 | 19193811 |
| 0114 | C | Incident | 3 | 0.06 | 0 | IV | - | SH | Naïve | 27 | 2003-4-24 | 19193811 |
| 0478 | C | Incident | 3 | 0.02 | 0 | I/II | - | SH | Naïve | 23 | 2003-8-15 | 19193811 |
| 1196 | C | Incident | 3 | 0.54 | 0 | I/II | - | SH | Naïve | 23 | 2004-6-15 | 19193811 |
| 1335 | C | Incident | 3 | 0.06 | 0 | IV | - | SH | Naïve | 21 | 2004-8-20 | 19193811 |
| 703010010 | C | Incident | 3 | 0.38 | 0 | III | - | SH | Naïve | 22 | 2006-12-28 | 19193811 |
| 703010200 | C | Incident | 3 | 0.00 | 0 | IV | - | SH | Naïve | 18 | 2007-5-18 | 19193811 |
| 703010228 | C | Incident | 2 | 0.03 | 0 | IV | - | SH | Naïve | 28 | 2007-6-7 | 19193811 |
| 706010151 | C | Incident | 2 | 0.91 | 0 | VI | - | SH | Naïve | 15 | 2007-8-23 | 19193811 |
| HOBR0961 | B | Incident | 1 | 0.03 | 0 | II | - | MSM | Naïve | 42 | 1991-10-31 | 20485520 |
| 4013419 | B | Incident | 3 | 0.03 | 0 | II | - | MSM | Naïve | 78 | 2006-3-14 | 20485520 |
| AD83 | B | Incident | 3 | 0.17 | 0 | V | - | MSM | Naïve | 44 | 2003-1-22 | 20485520 |

**Table S1**. **Detailed information of the dataset D561.**

| **Subject** | **Subtype** | **Defined*** | **T/Fs**** | **SCBD(%)** | **HD Q10** | **Fiebig Stage** | **Days post**  **Seroconversion** | **Risk***** | **HAART**  **Treat** | **Nseq** | **Sampling**  **Date** | **PMID** |
| --- | --- | --- | --- | --- | --- | --- | --- | --- | --- | --- | --- | --- |
| 701010055 | B | Incident | 1 | 0.01 | 0 | II | - | MSM | Naïve | 28 | 2006-10-5 | 20485520 |
| AD75 | B | Incident | 1 | 0.03 | 0 | II | - | MSM | Naïve | 54 | 2002-11-6 | 20485520 |
| AD17 | B | Incident | 1 | 0.03 | 0 | II | - | MSM | Naïve | 51 | 1999-6-14 | 20485520 |
| INME | B | Incident | 1 | 0.04 | 0 | II | - | MSM | Naïve | 46 | 1990-8-9 | 20485520 |
| AD77 | B | Incident | 1 | 0.21 | 0 | V | - | MSM | Naïve | 40 | 2002-11-15 | 20485520 |
| 4013242 | B | Incident | 1 | 0.06 | 0 | IV | - | MSM | Naïve | 37 | 2003-1-23 | 20485520 |
| 4013291 | B | Incident | 1 | 0.39 | 1 | V | - | MSM | Naïve | 25 | 2003-6-4 | 20485520 |
| 700010238 | B | Incident | 3 | 0.43 | 0 | V | - | MSM | Naïve | 38 | 2007-5-8 | 20485520 |
| 4013171 | B | Incident | >=10 | 0.10 | 0 | IV | - | MSM | Naïve | 86 | 2002-2-6 | 20485520 |
| 4013296 | B | Incident | 1 | 0.04 | 0 | II | - | MSM | Naïve | 25 | 2003-8-5 | 20485520 |
| 700010246 | B | Incident | 1 | 0.04 | 0 | IV | - | MSM | Naïve | 45 | 2007-6-7 | 20485520 |
| RIER | B | Chronic | - | 3.42 | 2 | - | - | MSM | Naïve | 23 | 1990 | 20485520 |
| 701010027 | B | Incident | 1 | 0.20 | 0 | V | - | MSM | Naïve | 27 | 2006-8-29 | 20485520 |
| 4013240 | B | Incident | 3 | 0.18 | 0 | II | - | MSM | Naïve | 66 | 2003-1-21 | 20485520 |
| 4013396 | B | Incident | 1 | 0.03 | 0 | IV | - | MSM | Naïve | 39 | 2005-8-16 | 20485520 |
| LACU9000 | B | Chronic | - | 1.97 | 7 | - | - | MSM | Naïve | 26 | 1991 | 20485520 |
| 4013226 | B | Incident | 1 | 0.11 | 0 | II | - | MSM | Naïve | 33 | 2002-11-20 | 20485520 |
| 4013448 | B | Incident | 4 | 0.04 | 0 | II | - | MSM | Naïve | 54 | 2007-1-19 | 20485520 |
| 4013327 | B | Incident | 1 | 0.03 | 0 | IV | - | MSM | Naïve | 24 | 2004-1-27 | 20485520 |
| 4013383 | B | Incident | 2 | 0.03 | 0 | II | - | MSM | Naïve | 69 | 2005-4-5 | 20485520 |
| 4013446 | B | Incident | 1 | 0.02 | 0 | III | - | MSM | Naïve | 23 | 2006-11-28 | 20485520 |
| 700010106 | B | Incident | 1 | 0.05 | 0 | II | - | MSM | Naïve | 40 | 2006-10-19 | 20485520 |
| 4013321 | B | Incident | 1 | 0.05 | 0 | II | - | MSM | Naïve | 49 | 2003-10-10 | 20485520 |

**Table S1**. **Detailed information of the dataset D561.**

| **Subject** | **Subtype** | **Defined*** | **T/Fs**** | **SCBD(%)** | **HD Q10** | **Fiebig Stage** | **Days post**  **Seroconversion** | **Risk***** | **HAART**  **Treat** | **Nseq** | **Sampling**  **Date** | **PMID** |
| --- | --- | --- | --- | --- | --- | --- | --- | --- | --- | --- | --- | --- |
| 4013211 | B | Incident | 2 | 0.05 | 0 | III | - | MSM | Naïve | 30 | 2002-8-23 | 20485520 |
| 701010108 | B | Incident | 1 | 0.04 | 0 | V | - | MSM | Naïve | 35 | 2007-6-28 | 20485520 |
| 701010068 | B | Incident | 7 | 0.05 | 0 | IV | - | MSM | Naïve | 89 | 2006-10-24 | 20485520 |
| 4013440 | B | Incident | 1 | 0.05 | 0 | II | - | MSM | Naïve | 29 | 2006-10-17 | 20485520 |
| 9025 | B | Incident | 1 | 0.13 | 0 | - | - | - | Naïve | 19 | 2008-4-21 | 20015984 |
| 7146 | B | Incident | 2 | 0.51 | 0 | - | - | - | Naïve | 36 | 2003-10-14 | 20015984 |
| 9039 | B | Incident | 1 | 0.05 | 0 | - | - | - | Naïve | 40 | 2009-6-1 | 20015984 |
| 9007 | B | Chronic | - | 1.15 | 1 | - | - | - | Naïve | 20 | 2007-6-5 | 20015984 |
| 9040 | B | Incident | 1 | 0.57 | 1 | - | - | - | Naïve | 23 | 2009-6-15 | 20015984 |
| 7146 | B | Incident | 2 | 0.35 | 0 | - | - | - | Naïve | 22 | 2003-9-23 | 20015984 |
| 9037 | B | Incident | 1 | 0.09 | 0 | - | - | - | Naïve | 37 | 2009-5-14 | 20015984 |
| 7146 | B | Chronic | - | 0.89 | 0 | - | - | - | Treated | 33 | 2004-10-7 | 20015984 |
| 9018 | B | Incident | >=2 | 0.52 | 0 | - | - | - | Naïve | 29 | 2007-8-2 | 20015984 |
| 9002 | B | Incident | 1 | 0.93 | 3 | - | - | - | Naïve | 20 | 2006-10-26 | 20015984 |
| 7146 | B | Incident | >1 | 0.17 | 0 | - | - | - | Naïve | 26 | 2003-11-10 | 20015984 |
| 9007 | B | Incident | 1 | 0.35 | 0 | - | - | - | Naïve | 29 | 2006-9-18 | 20015984 |
| R526 | A1 | Incident | 1 | 0.01 | 0 | I/II | - | IDU | Naïve | 28 | 2007 | 20423223 |
| R163 | A1 | Incident | 1 | 0.00 | 0 | I/II | - | IDU | Naïve | 21 | 2006 | 20423223 |
| R497 | A1 | Incident | 1 | 0.29 | 1 | VI | - | IDU | Naïve | 25 | 2007 | 20423223 |
| R526 | A1 | Incident | 1 | 0.18 | 0 | V | - | IDU | Naïve | 19 | 2007 | 20423223 |
| K84 | A1 | Incident | 1 | 0.07 | 0 | V | - | IDU | Naïve | 23 | 2008 | 20423223 |
| SC1457 | A1 | Chronic | - | 3.96 | 4 | VI | - | IDU | Naïve | 21 | 2006 | 20423223 |
| R575 | A1 | Incident | 1 | 0.10 | 0 | IV | - | IDU | Naïve | 18 | 2007 | 20423223 |

**Table S1**. **Detailed information of the dataset D561.**

| **Subject** | **Subtype** | **Defined*** | **T/Fs**** | **SCBD(%)** | **HD Q10** | **Fiebig Stage** | **Days post**  **Seroconversion** | **Risk***** | **HAART**  **Treat** | **Nseq** | **Sampling**  **Date** | **PMID** |
| --- | --- | --- | --- | --- | --- | --- | --- | --- | --- | --- | --- | --- |
| SC3208 | A1 | Chronic | - | 2.40 | 5 | VI | - | IDU | Naïve | 25 | 2007 | 20423223 |
| H386 | A1 | Incident | 1 | 0.09 | 0 | IV | - | IDU | Naïve | 26 | 2008 | 20423223 |
| R589 | A1 | Incident | 1 | 0.13 | 0 | VI | - | IDU | Naïve | 29 | 2008 | 20423223 |
| H386 | A1 | Incident | 1 | 0.08 | 0 | V | - | IDU | Naïve | 23 | 2008 | 20423223 |
| H408 | A1 | Incident | 2 | 0.12 | 0 | IV | - | IDU | Naïve | 19 | 2002 | 20423223 |
| SC1233 | A1 | Chronic | - | 1.20 | 1 | VI | - | IDU | Naïve | 25 | 2006 | 20423223 |
| SC3410 | A1 | Chronic | - | 1.67 | 2 | VI | - | IDU | Naïve | 23 | 2008 | 20423223 |
| R053 | A1 | Incident | >2 | 0.13 | 0 | IV | - | IDU | Naïve | 22 | 2008 | 20423223 |
| R497 | A1 | Incident | 1 | 0.09 | 0 | IV | - | IDU | Naïve | 26 | 2007 | 20423223 |
| R053 | A1 | Incident | >2 | 0.22 | 0 | V | - | IDU | Naïve | 20 | 2008 | 20423223 |
| H410 | A1 | Incident | 1 | 0.26 | 0 | III | - | IDU | Naïve | 27 | 2002 | 20423223 |
| SC1283 | 06A1 | Incident | 2 | 0.58 | 1 | VI | - | IDU | Naïve | 24 | 2006 | 20423223 |
| K84 | A1 | Incident | 1 | 0.08 | 0 | IV | - | IDU | Naïve | 29 | 2008 | 20423223 |
| H410 | A1 | Incident | 3 | 0.28 | 0 | IV | - | IDU | Naïve | 24 | 2002 | 20423223 |
| H408 | A1 | Incident | 2 | 0.07 | 0 | III | - | IDU | Naïve | 27 | 2002 | 20423223 |
| R392 | A1 | Incident | 1 | 0.04 | 0 | IV | - | IDU | Naïve | 29 | 2006 | 20423223 |
| K08 | A1 | Incident | 1 | 0.36 | 0 | VI | - | IDU | Naïve | 23 | 2008 | 20423223 |
| ZM178F | C | Incident | 1 | 0.53 | 1 | VI | - | SH | Naïve | 26 | 2003-1-18 | 18256145 |
| ZM215F | C | Incident | >=4 | 0.08 | 0 | VI | - | SH | Naïve | 32 | 2002-10-19 | 18256145 |
| ZM206F | C | Incident | 1 | 0.34 | 0 | VI | - | SH | Naïve | 35 | 2002-7-13 | 18256145 |
| ZM246F | C | Incident | 1 | 0.49 | 1 | VI | - | SH | Naïve | 14 | 2003-4-4 | 18256145 |
| ZM249M | C | Incident | 1 | 0.07 | 0 | V | - | SH | Naïve | 25 | 2003-8-12 | 18256145 |
| ZM247F | C | Incident | 2 | 0.19 | 0 | III | - | SH | Naïve | 19 | 2003-11-1 | 18256145 |

**Table S1**. **Detailed information of the dataset D561.**

| **Subject** | **Subtype** | **Defined*** | **T/Fs**** | **SCBD(%)** | **HD Q10** | **Fiebig Stage** | **Days post**  **Seroconversion** | **Risk***** | **HAART**  **Treat** | **Nseq** | **Sampling**  **Date** | **PMID** |
| --- | --- | --- | --- | --- | --- | --- | --- | --- | --- | --- | --- | --- |
| ZM249M | C | Incident | 1 | 0.02 | 0 | IV | - | SH | Naïve | 24 | 2003-8-5 | 18256145 |
| ZM184F | C | Incident | 1 | 0.15 | 0 | V | - | SH | Naïve | 26 | 2003-7-10 | 18256145 |
| ZM214M | C | Incident | >1 | 0.71 | 1 | VI | - | SH | Naïve | 35 | 2003-7-2 | 18256145 |
| ZM247F | C | Incident | 2 | 0.37 | 0 | II | - | SH | Naïve | 25 | 2003-10-28 | 18256145 |
| ZM235F | C | Incident | 1 | 0.08 | 0 | V | - | SH | Naïve | 28 | 2002-12-20 | 18256145 |
| ZM180M | C | Incident | 1 | 0.17 | 0 | V | - | SH | Naïve | 24 | 2002-7-20 | 18256145 |
| ZM229M | C | Incident | >=4 | 0.30 | 1 | VI | - | SH | Naïve | 29 | 2002-10-19 | 18256145 |
| ZM231F | CF1U | Incident | 1 | 0.31 | 0 | V | - | SH | Naïve | 48 | 2002-12-17 | 18256145 |
| ZM246F | C | Incident | 1 | 0.08 | 0 | II | - | SH | Naïve | 27 | 2003-1-14 | 18256145 |
| 1002 | B | Chronic | - | 1.90 | 3 | - | - | SH | Naïve | 31 | 2006 | 19339344 |
| 1013 | B | Chronic | - | 3.14 | 9 | - | - | - | Treated | 44 | 2007 | 19339344 |
| 1025 | D | Chronic | - | 2.96 | 8 | - | - | - | Treated | 24 | 2007 | 19339344 |
| 1004 | 01_AE | Chronic | - | 1.71 | 1 | - | - | SH | Treated | 40 | 2006 | 19339344 |
| 1009 | B | Chronic | - | 2.57 | 6 | - | - | SH | Treated | 27 | 2006 | 19339344 |
| 1010 | A1 | Chronic | - | 2.32 | 2 | - | - | SH | Naïve | 22 | 2006 | 19339344 |
| 1021 | B | Chronic | - | 3.46 | 14 | - | - | - | Treated | 21 | 2007 | 19339344 |
| 1014 | B | Chronic | - | 2.94 | 4 | - | - | SH | Naïve | 30 | 2006 | 19339344 |
| 3312 | C | Incident | 1 | 0.26 | 1 | - | 197 | NR | Naïve | 13 | 2006 | 18973914 |
| 5018 | C | Incident | >1 | 0.95 | 4 | - | 203 | NR | Naïve | 7 | 2007 | 18973914 |
| 1811 | C | Incident | >1 | 0.97 | 0 | - | 16 | NR | Naïve | 10 | 2004 | 18973914 |
| 3505 | C | Chronic | - | 1.41 | 0 | - | 440 | NR | Naïve | 5 | 2007 | 18973914 |
| 3430 | C | Incident | 1 | 0.03 | 0 | - | 275 | NR | Naïve | 12 | 2006 | 18973914 |
| 3603 | C | Incident | 1 | 0.36 | 0 | - | 341 | NR | Naïve | 6 | 2007 | 18973914 |

**Table S1**. **Detailed information of the dataset D561.**

| **Subject** | **Subtype** | **Defined*** | **T/Fs**** | **SCBD(%)** | **HD Q10** | **Fiebig Stage** | **Days post**  **Seroconversion** | **Risk***** | **HAART**  **Treat** | **Nseq** | **Sampling**  **Date** | **PMID** |
| --- | --- | --- | --- | --- | --- | --- | --- | --- | --- | --- | --- | --- |
| 3505 | C | Incident | 1 | 1.15 | 4 | - | 316 | NR | Naïve | 6 | 2007 | 18973914 |
| 3505 | C | Incident | 1 | 0.88 | 2 | - | 196 | NR | Naïve | 7 | 2006 | 18973914 |
| 2865 | C | Incident | 1 | 0.72 | 1 | - | 78 | NR | Naïve | 13 | 2005 | 18973914 |
| 3312 | C | Chronic | - | 1.54 | 2 | - | 441 | NR | Naïve | 7 | 2007 | 18973914 |
| 5018 | C | Incident | >1 | 0.52 | 2 | - | 295 | NR | Naïve | 7 | 2007 | 18973914 |
| 2865 | C | Incident | 1 | 0.96 | 2 | - | 154 | NR | Naïve | 11 | 2006 | 18973914 |
| 5582 | C | Incident | 1 | 0.00 | 0 | - | 44 | NR | Naïve | 13 | 2007 | 18973914 |
| 1811 | C | Incident | >1 | 0.58 | 1 | - | 91 | NR | Naïve | 17 | 2004 | 18973914 |
| 2865 | C | Incident | 1 | 0.22 | 0 | - | 0 | NR | Naïve | 11 | 2005 | 18973914 |
| 3312 | C | Incident | 1 | 0.32 | 0 | - | 288 | NR | Naïve | 15 | 2006 | 18973914 |
| 5018 | C | Chronic | - | 2.40 | 6 | - | 387 | NR | Naïve | 6 | 2007 | 18973914 |
| 5582 | C | Incident | 1 | 0.06 | 0 | - | 87 | NR | Naïve | 13 | 2007 | 18973914 |
| 5018 | C | Incident | >1 | 0.46 | 0 | - | 0 | NR | Naïve | 22 | 2006 | 18973914 |
| 3430 | C | Incident | 1 | 0.36 | 0 | - | 244 | NR | Naïve | 8 | 2006 | 18973914 |
| 3430 | C | Chronic | - | 0.31 | 1 | - | 374 | NR | Naïve | 10 | 2007 | 18973914 |
| 5582 | C | Incident | 1 | 0.40 | 1 | - | 336 | NR | Naïve | 10 | 2008 | 18973914 |
| 2865 | C | Incident | 1 | 0.74 | 2 | - | 213 | NR | ART | 13 | 2006 | 18973914 |
| 3430 | C | Incident | 1 | 0.13 | 0 | - | 29 | NR | Naïve | 12 | 2006 | 18973914 |
| 1811 | C | Chronic | - | 2.15 | 1 | - | 350 | NR | ART | 12 | 2005 | 18973914 |
| 3312 | C | Incident | 1 | 0.45 | 1 | - | 105 | NR | Naïve | 6 | 2006 | 18973914 |
| 3603 | C | Incident | 1 | 0.20 | 0 | - | 193 | NR | Naïve | 9 | 2007 | 18973914 |
| 5018 | C | Incident | >1 | 0.10 | 0 | - | 97 | NR | Naïve | 7 | 2007 | 18973914 |
| 3430 | C | Incident | 1 | 0.42 | 0 | - | 0 | NR | Naïve | 12 | 2006 | 18973914 |

**Table S1**. **Detailed information of the dataset D561.**

| **Subject** | **Subtype** | **Defined*** | **T/Fs**** | **SCBD(%)** | **HD Q10** | **Fiebig Stage** | **Days post**  **Seroconversion** | **Risk***** | **HAART**  **Treat** | **Nseq** | **Sampling**  **Date** | **PMID** |
| --- | --- | --- | --- | --- | --- | --- | --- | --- | --- | --- | --- | --- |
| 5582 | C | Incident | 1 | 0.36 | 0 | - | 260 | NR | Naïve | 11 | 2008 | 18973914 |
| 5582 | C | Incident | 1 | 0.48 | 1 | - | 191 | NR | Naïve | 7 | 2007 | 18973914 |
| 5582 | C | Incident | 1 | 0.00 | 0 | - | 5 | NR | Naïve | 12 | 2007 | 18973914 |
| 3312 | C | Incident | 1 | 0.29 | 0 | - | 0 | NR | Naïve | 14 | 2005 | 18973914 |
| 3603 | C | Incident | 1 | 0.36 | 1 | - | 291 | NR | Naïve | 9 | 2007 | 18973914 |
| 3430 | C | Incident | 1 | 0.66 | 0 | - | 120 | NR | Naïve | 12 | 2006 | 18973914 |
| 1811 | C | Incident | >1 | 0.59 | 1 | - | 260 | NR | Naïve | 11 | 2004 | 18973914 |
| 3603 | C | Incident | 1 | 0.00 | 0 | - | 0 | NR | ART | 11 | 2006 | 18973914 |
| 3603 | C | Incident | 1 | 0.07 | 0 | - | 91 | NR | Naïve | 11 | 2006 | 18973914 |
| 5018 | C | Incident | >1 | 0.18 | 0 | - | 171 | NR | Naïve | 8 | 2007 | 18973914 |
| 3505 | C | Incident | 1 | 0.06 | 0 | - | 0 | NR | Naïve | 13 | 2006 | 18973914 |
| 3505 | C | Incident | 1 | 0.08 | 0 | - | 63 | NR | Naïve | 15 | 2006 | 18973914 |
| 3430 | C | Incident | 1 | 0.17 | 0 | - | 309 | NR | Naïve | 7 | 2006 | 18973914 |
| TT27 | B | Incident | 2 | 0.42 | 0 | - | 5 | SH | Naïve | 20 | 1998 | 21698149 |
| SC33 | B | Incident | 2 | 0.94 | 0 | - | 43 | SH | Naïve | 19 | 1994 | 21698149 |
| 145447 | B | Incident | 1 | 0.19 | 0 | - | 90 | MSM | Naïve | 10 | 2003 | 21698149 |
| SC24 | B | Incident | 1 | 0.04 | 0 | - | 34 | SH | Naïve | 19 | 1994 | 21698149 |
| SC22 | B | Incident | 1 | 0.00 | 0 | - | 35 | SH | Naïve | 15 | 1994 | 21698149 |
| 159687 | B | Incident | 1 | 0.16 | 0 | - | 90 | MSM | Naïve | 9 | 2004 | 21698149 |
| 151263 | B | Incident | 1 | 0.07 | 0 | - | 90 | MSM | Naïve | 20 | 2003 | 21698149 |
| SC34 | B | Incident | 1 | 0.15 | 0 | - | 6 | SH | Naïve | 12 | 1994 | 21698149 |
| TT34 | B | Incident | 1 | 0.00 | 0 | - | 13 | SH | Naïve | 13 | 1999 | 21698149 |
| SC45 | B | Incident | 1 | 0.19 | 0 | - | 46 | SH | Naïve | 20 | 1995 | 21698149 |

**Table S1**. **Detailed information of the dataset D561.**

| **Subject** | **Subtype** | **Defined*** | **T/Fs**** | **SCBD(%)** | **HD Q10** | **Fiebig Stage** | **Days post**  **Seroconversion** | **Risk***** | **HAART**  **Treat** | **Nseq** | **Sampling**  **Date** | **PMID** |
| --- | --- | --- | --- | --- | --- | --- | --- | --- | --- | --- | --- | --- |
| TT31 | B | Incident | 1 | 0.16 | 0 | - | 5 | SH | Naïve | 19 | 1998 | 21698149 |
| SC20 | B | Incident | 1 | 0.04 | 0 | - | 26 | SH | Naïve | 20 | 1994 | 21698149 |
| SC25 | B | Incident | 1 | 0.40 | 1 | - | 59 | SH | Naïve | 19 | 1994 | 21698149 |
| SC41 | B | Incident | 1 | 0.09 | 0 | - | 40 | SH | Naïve | 20 | 1995 | 21698149 |
| SC11 | B | Incident | 1 | 0.27 | 0 | - | 37 | SH | Naïve | 7 | 1993 | 21698149 |
| 137681 | B | Incident | 1 | 0.37 | 0 | - | 90 | MSM | Naïve | 20 | 2003 | 21698149 |
| 142467 | B | Incident | 1 | 0.15 | 0 | - | 90 | MSM | Naïve | 11 | 2003 | 21698149 |
| TT37 | B | Incident | 3 | 0.06 | 0 | - | 0 | SH | Naïve | 8 | 2000 | 21698149 |
| SC47 | B | Incident | 2 | 0.16 | 0 | - | 30 | SH | Naïve | 19 | 1994 | 21698149 |
| 134742 | B | Incident | 1 | 0.19 | 0 | - | 90 | MSM | Naïve | 14 | 2003 | 21698149 |
| SC42 | B | Incident | >=2 | 0.44 | 0 | - | 7 | SH | Naïve | 20 | 1995 | 21698149 |
| SC31 | B | Incident | 1 | 0.06 | 0 | - | 25 | SH | Naïve | 20 | 1994 | 21698149 |
| TT28 | B | Incident | 1 | 0.13 | 0 | - | 3 | SH | Naïve | 20 | 1994 | 21698149 |
| 138910 | B | Incident | 1 | 0.10 | 0 | - | 60 | MSM | Naïve | 20 | 2003 | 21698149 |
| 101815 | B | Incident | 2 | 0.47 | 0.5 | - | 75 | MSM | Naïve | 5 | 2001 | 21698149 |
| SC05 | B | Incident | 1 | 0.02 | 0 | - | 10 | SH | Naïve | 19 | 1993 | 21698149 |
| SC35 | B | Incident | 1 | 0.11 | 0 | - | 34 | SH | Naïve | 20 | 1994 | 21698149 |
| 1001 | B | Incident | 5 | 0.46 | 1 | V | - | IDU | Naïve | 25 | 1998 | 20375173 |
| CQLDR03 | B | Incident | 1 | 0.11 | 0 | V | - | IDU | Naïve | 22 | 1997 | 20375173 |
| PSL024 | B | Incident | 3 | 0.26 | 2 | VI | - | IDU | Naïve | 19 | 2003 | 20375173 |
| 1029 | B | Incident | 1 | 0.08 | 0 | IV | - | IDU | Naïve | 28 | 2003 | 20375173 |
| 1034 | B | Incident | 16 | 0.86 | 0 | III | - | IDU | Naïve | 166 | 2004 | 20375173 |
| 54869022 | B | Incident | 1 | 0.09 | 0 | VI | - | IDU | Naïve | 26 | 1998 | 20375173 |

**Table S1**. **Detailed information of the dataset D561.**

| **Subject** | **Subtype** | **Defined*** | **T/Fs**** | **SCBD(%)** | **HD Q10** | **Fiebig Stage** | **Days post**  **Seroconversion** | **Risk***** | **HAART**  **Treat** | **Nseq** | **Sampling**  **Date** | **PMID** |
| --- | --- | --- | --- | --- | --- | --- | --- | --- | --- | --- | --- | --- |
| 1032 | B | Incident | 3 | 0.04 | 0 | V | - | IDU | Naïve | 19 | 2003 | 20375173 |
| HTM385 | B | Incident | 1 | 0.03 | 0 | V | - | IDU | Naïve | 22 | 2006 | 20375173 |
| HTM319 | B | Incident | 3 | 0.54 | 1 | V | - | IDU | Naïve | 31 | 1997 | 20375173 |
| 580208 | B | Incident | 3 | 1.00 | 1 | VI | - | IDU | Naïve | 30 | 1997 | 20375173 |
| 398 | A1 | Incident | 1 | 0.21 | 0 | - | - | SH | Naïve | 7 | 2000 | 21531432 |
| 605 | A1C | Incident | 1 | 0.04 | 0 | V/VI | - | SH | Naïve | 9 | 2000 | 21531432 |
| 21 | A | Incident | >1 | 0.25 | 0 | V/VI | - | SH | Naïve | 11 | 2000 | 21531432 |
| 234 | C | Incident | 1 | 0.00 | 0 | V/VI | - | SH | Naïve | 12 | 2000 | 21531432 |
| 142 | C | Incident | >1 | 0.38 | 1 | VI | - | SH | Naïve | 9 | 2000 | 21531432 |
| 346 | C | Incident | 1 | 0.24 | 0 | VI | - | SH | Naïve | 8 | 2000 | 21531432 |
| 401 | CD | Incident | 1 | 0.06 | 0 | - | - | SH | Naïve | 6 | 2000 | 21531432 |
| 532 | A1 | Incident | >1 | 0.00 | 0 | I/II | - | SH | Naïve | 8 | 2000 | 21531432 |
| 541 | C | Incident | 1 | 0.06 | 0 | VI | - | SH | Naïve | 11 | 2000 | 21531432 |
| 515 | A1 | Incident | 1 | 0.22 | 0.5 | VI | - | SH | Naïve | 5 | 2000 | 21531432 |
| 410 | A1C | Incident | 2 | 0.21 | 0 | VI | - | SH | Naïve | 11 | 2000 | 21531432 |
| 246 | A1C | Incident | 1 | 0.06 | 0 | VI | - | SH | Naïve | 18 | 2000 | 21531432 |
| 304 | C | Incident | 1 | 0.03 | 0 | V | - | SH | Naïve | 11 | 2000 | 21531432 |
| 390 | C | Incident | 1 | 0.12 | 0 | V | - | SH | Naïve | 11 | 2000 | 21531432 |
| 569 | C | Incident | 1 | 0.04 | 0 | V/VI | - | SH | Naïve | 11 | 2000 | 21531432 |
| 98 | C | Incident | >1 | 0.62 | 0 | V | - | SH | Naïve | 6 | 2000 | 21531432 |
| 556 | C | Incident | >1 | 0.27 | 0 | V | - | SH | Naïve | 11 | 2000 | 21531432 |
| 49 | ACD | Incident | 1 | 0.00 | 0 | III | - | SH | Naïve | 10 | 2000 | 21531432 |
| 89 | CD | Incident | 1 | 0.00 | 0 | I/II | - | SH | Naïve | 5 | 2000 | 21531432 |

**Table S1**. **Detailed information of the dataset D561.**

| **Subject** | **Subtype** | **Defined*** | **T/Fs**** | **SCBD(%)** | **HD Q10** | **Fiebig Stage** | **Days post**  **Seroconversion** | **Risk***** | **HAART**  **Treat** | **Nseq** | **Sampling**  **Date** | **PMID** |
| --- | --- | --- | --- | --- | --- | --- | --- | --- | --- | --- | --- | --- |
| 216 | A1 | Incident | 1 | 0.00 | 0 | VI | - | SH | Naïve | 8 | 2000 | 21531432 |
| 477 | A1C | Incident | 1 | 0.87 | 1 | VI | - | SH | Naïve | 11 | 2000 | 21531432 |
| 54 | D | Incident | 1 | 0.00 | 0 | VI | - | SH | Naïve | 9 | 2000 | 21531432 |
| 700010077 | B | Incident | 1 | 0.62 | 1.5 | - | 102 | MSM | Naïve | 5 | 2006 | 21347345 |
| 700010040 | B | Incident | 1 | 0.07 | 0 | - | 45 | MSM | Naïve | 31 | 2006 | 21347345 |
| 700010058 | B | Incident | 1 | 0.21 | 0 | - | 85 | - | Naïve | 9 | 2006 | 21347345 |
| 700010058 | B | Incident | 1 | 0.00 | 0 | - | 45 | - | Naïve | 9 | 2006 | 21347345 |
| 700010040 | B | Incident | 1 | 0.30 | 0 | - | 111 | MSM | Naïve | 9 | 2006 | 21347345 |
| 700010040 | B | Incident | 1 | 0.48 | 0 | - | 181 | MSM | Naïve | 11 | 2007 | 21347345 |
| 700010040 | B | Chronic | - | 1.17 | 1 | - | 412 | MSM | Naïve | 12 | 2007 | 21347345 |
| 700010077 | B | Incident | 1 | 0.48 | 1 | - | 159 | MSM | Naïve | 12 | 2007 | 21347345 |
| 700010077 | B | Incident | 1 | 0.00 | 0 | - | 32 | MSM | Naïve | 12 | 2006 | 21347345 |
| ZM185F | C | Chronic | - | 2.63 | 8 | - | 856 | SH | Naïve | 10 | 2004 | 19763269 |
| ZM185F | C | Incident | 1 | 0.85 | 2 | - | 262 | SH | Naïve | 10 | 2003 | 19763269 |
| ZM185F | C | Incident | 1 | 0.39 | 0.5 | - | 18 | SH | Naïve | 5 | 2002 | 19763269 |
| ZM185F | C | Chronic | - | 1.45 | 2 | - | 443 | SH | Naïve | 10 | 2003 | 19763269 |
| ZM205F | C | Chronic | - | 1.04 | 1.5 | - | 439 | SH | Naïve | 5 | 2004 | 19763269 |
| ZM185F | C | Incident | 1 | 0.45 | 0 | - | 177 | SH | Naïve | 9 | 2003 | 19763269 |
| ZM205F | C | Chronic | - | 1.66 | 6 | - | 615 | SH | Naïve | 5 | 2004 | 19763269 |
| ZM185F | C | Chronic | - | 1.83 | 2 | - | 704 | SH | Naïve | 9 | 2004 | 19763269 |
| ZM185F | C | Chronic | - | 2.27 | 6 | - | 620 | SH | Naïve | 8 | 2004 | 19763269 |
| ZM205F | C | Incident | 1 | 0.16 | 0 | - | 26 | SH | Naïve | 5 | 2003 | 19763269 |
| ZM185F | C | Incident | 1 | 0.08 | 0 | - | 11 | SH | Naïve | 5 | 2002 | 19763269 |

**Table S1**. **Detailed information of the dataset D561.**

| **Subject** | **Subtype** | **Defined*** | **T/Fs**** | **SCBD(%)** | **HD Q10** | **Fiebig Stage** | **Days post**  **Seroconversion** | **Risk***** | **HAART**  **Treat** | **Nseq** | **Sampling**  **Date** | **PMID** |
| --- | --- | --- | --- | --- | --- | --- | --- | --- | --- | --- | --- | --- |
| ZM185F | C | Incident | 1 | 0.15 | 0 | - | 81 | SH | Naïve | 10 | 2002 | 19763269 |
| ZM205F | C | Incident | 1 | 0.64 | 2 | - | 258 | SH | Naïve | 5 | 2003 | 19763269 |
| ZM185F | C | Chronic | - | 1.38 | 4 | - | 529 | SH | Naïve | 9 | 2004 | 19763269 |
| ZM185F | C | Chronic | - | 1.77 | 3 | - | 787 | SH | Naïve | 8 | 2004 | 19763269 |
| ZM205F | C | Chronic | - | 1.44 | 5 | - | 1167 | SH | Naïve | 5 | 2006 | 19763269 |
| ZM185F | C | Chronic | - | 1.12 | 2 | - | 359 | SH | Naïve | 10 | 2003 | 19763269 |
| ZM417 | C | Chronic | - | 2.27 | 0 | - | - | SH | Naïve | 9 | 2005 | 21036380 |
| ZM412 | C | Chronic | - | 3.30 | 7 | - | - | SH | Naïve | 10 | 2005 | 21036380 |
| ZM410 | C | Chronic | - | 3.97 | 21 | - | - | SH | Naïve | 11 | 2005 | 21036380 |
| ZM402 | C | Chronic | - | 3.99 | 10 | - | - | SH | Naïve | 10 | 2005 | 21036380 |
| ZM373 | C | Chronic | - | 2.68 | 12 | - | - | SH | Naïve | 5 | 2005 | 21036380 |
| ZM406 | C | Chronic | - | 2.61 | 4 | - | - | SH | Naïve | 10 | 2005 | 21036380 |
| ZM403 | C | Chronic | - | 3.81 | 12 | - | - | SH | Naïve | 11 | 2005 | 21036380 |
| ZM389 | C | Chronic | - | 4.37 | 13 | - | - | SH | Naïve | 10 | 2005 | 21036380 |
| ZM416 | C | Chronic | - | 3.46 | 5 | - | - | SH | Naïve | 23 | 2005 | 21036380 |
| ZM419 | C | Chronic | - | 1.24 | 1 | - | - | SH | Naïve | 10 | 2005 | 21036380 |
| ZM384 | C | Chronic | - | 2.90 | 3 | - | - | SH | Naïve | 11 | 2005 | 21036380 |
| ZM387 | D | Chronic | - | 3.75 | 24 | - | - | SH | Naïve | 9 | 2005 | 21036380 |
| ZM411 | C | Chronic | - | 1.72 | 4 | - | - | SH | Naïve | 14 | 2005 | 21036380 |
| ZM420 | C | Chronic | - | 4.02 | 8 | - | - | SH | Naïve | 14 | 2005 | 21036380 |
| ZM379 | C | Chronic | - | 1.97 | 5 | - | - | SH | Naïve | 14 | 2005 | 21036380 |
| ZM408 | C | Chronic | - | 5.34 | 5 | - | - | SH | Naïve | 10 | 2005 | 21036380 |
| ZM394 | C | Chronic | - | 3.83 | 5 | - | - | SH | Naïve | 20 | 2005 | 21036380 |

**Table S1**. **Detailed information of the dataset D561.**

| **Subject** | **Subtype** | **Defined*** | **T/Fs**** | **SCBD(%)** | **HD Q10** | **Fiebig Stage** | **Days post**  **Seroconversion** | **Risk***** | **HAART**  **Treat** | **Nseq** | **Sampling**  **Date** | **PMID** |
| --- | --- | --- | --- | --- | --- | --- | --- | --- | --- | --- | --- | --- |
| ZM378 | C | Chronic | - | 5.38 | 6 | - | - | SH | Naïve | 13 | 2005 | 21036380 |
| ZM388 | C | Chronic | - | 1.83 | 7 | - | - | SH | Naïve | 11 | 2005 | 21036380 |
| ZM376 | C | Chronic | - | 4.87 | 10 | - | - | SH | Naïve | 15 | 2005 | 21036380 |
| ZM399 | C | Chronic | - | 2.31 | 3 | - | - | SH | Naïve | 14 | 2005 | 21036380 |
| ZM381 | C | Chronic | - | 1.96 | 9 | - | - | SH | Naïve | 13 | 2005 | 21036380 |
| ZM400 | C | Chronic | - | 3.20 | 9 | - | - | SH | Naïve | 17 | 2005 | 21036380 |
| ZM415 | C | Chronic | - | 5.17 | 1.5 | - | - | SH | Naïve | 21 | 2005 | 21036380 |
| ZM377 | C | Chronic | - | 3.65 | 5.5 | - | - | SH | Naïve | 16 | 2005 | 21036380 |
| ZM393 | C | Chronic | - | 1.38 | 5 | - | - | SH | Naïve | 11 | 2005 | 21036380 |
| ZM375 | C | Chronic | - | 3.41 | 6 | - | - | SH | Naïve | 10 | 2005 | 21036380 |
| ZM382 | C | Chronic | - | 3.73 | 4 | - | - | SH | Naïve | 10 | 2005 | 21036380 |
| ZM418 | C | Chronic | - | 2.80 | 4 | - | - | SH | Naïve | 10 | 2005 | 21036380 |
| ZM380 | A1C | Chronic | - | 2.02 | 6 | - | - | SH | Naïve | 10 | 2005 | 21036380 |
| ZM383 | C | Chronic | - | 2.53 | 8 | - | - | SH | Naïve | 10 | 2005 | 21036380 |
| ZM401 | C | Chronic | - | 5.22 | 1 | - | - | SH | Naïve | 11 | 2005 | 21036380 |
| ZM405 | C | Chronic | - | 2.94 | 4 | - | - | SH | Naïve | 12 | 2005 | 21036380 |
| ZM395 | C | Chronic | - | 2.92 | 3 | - | - | SH | Naïve | 21 | 2005 | 21036380 |
| ZM407 | GJ | Chronic | - | 1.35 | 3 | - | - | SH | Naïve | 11 | 2005 | 21036380 |
| ZM413 | C | Chronic | - | 1.32 | 4 | - | - | SH | Naïve | 16 | 2005 | 21036380 |
| ZM414 | C | Chronic | - | 4.79 | 2 | - | - | SH | Naïve | 22 | 2005 | 21036380 |
| P1 | B | Incident | 1 | 0.14 | 0 | - | 38 | MSM | Naïve | 21 | 2004 | 21736738 |
| P2 | B | Incident | 1 | 0.44 | 0 | - | 179 | MSM | Naïve | 16 | 2005 | 21736738 |
| P2 | B | Incident | 1 | 0.07 | 0 | - | 7 | MSM | Naïve | 19 | 2004 | 21736738 |

**Table S1**. **Detailed information of the dataset D561.**

| **Subject** | **Subtype** | **Defined*** | **T/Fs**** | **SCBD(%)** | **HD Q10** | **Fiebig Stage** | **Days post**  **Seroconversion** | **Risk***** | **HAART**  **Treat** | **Nseq** | **Sampling**  **Date** | **PMID** |
| --- | --- | --- | --- | --- | --- | --- | --- | --- | --- | --- | --- | --- |
| P1 | B | Incident | >=3 | 0.05 | 0 | - | 210 | MSM | Naïve | 13 | 2005 | 21736738 |
| SK221 | C | Chronic | - | 3.02 | 3.5 | - | - | - | - | 16 | - | 21050445 |
| SK010 | C | Chronic | - | 2.16 | 6 | - | - | SH | - | 22 | - | 21050445 |
| SK312 | C | Chronic | - | 3.95 | 7 | - | - | - | - | 11 | - | 21050445 |
| SK200 | C | Chronic | - | 2.12 | 0 | - | - | SH | - | 20 | - | 21050445 |
| SK035 | C | Chronic | - | 3.90 | 6 | - | - | - | - | 23 | - | 21050445 |
| SK169 | C | Chronic | - | 5.20 | 10 | - | - | SH | - | 21 | - | 21050445 |
| SK233 | C | Chronic | - | 1.86 | 1 | - | - | SH | - | 18 | - | 21050445 |
| SK036 | C | Chronic | - | 3.69 | 4 | - | - | SH | - | 14 | - | 21050445 |

*Defined: Samples are defined as incident or chronic infections were identified as incident or chronic infections using combined information from the original sources, including Fiebig stage, clinical records of time since diagnosis (incident infection defined as under one year), and/or symptoms of acute infection.

**T/Fs: Number of estimated transmitted/founder (T/F) strains obtained from original sources.

***Risk: Risk behavior obtained from original sources. Subjects listed as "SH" were infected from heterosexual transmission; listed as “MSM” were transmitted from male-to-male; listed as “IDU” were injection drug users; otherwise, unknown transmission were considered.
